# Supplementary material for: Alternative splicing of PBRM1 mediates resistance to PD-1 blockade therapy in renal cancer
Source: EMBO J. 2024 Oct 7;43(22):7. doi: 10.1038/s44318-024-00262-7 (PMC11574163; doi:10.1038/s44318-024-00262-7)
Supplement: Supplementary file 3 — Appendix [file 44318_2024_262_MOESM3_ESM.pdf]

APPENDIX

Alternative splicing of PBRM1 mediates resistance to PD-1 blockade therapy in renal cancer

Namjoon Cho, Seung-Yeon Kim, Sung-Gwon Lee, Chungoo Park, Sunkyung Choi, Eun-Mi Kim, and Kee K. Kim

Table of Contents:

Appendix Figure S1 Comprehensive AS analysis in key cancer deriver genes.

Appendix Figure S2 AS pattern of *PBRM1* in cancer tissues.

Appendix Figure S3 Predicted secondary structure of the E27 region in PBRM1 splicing isoforms.

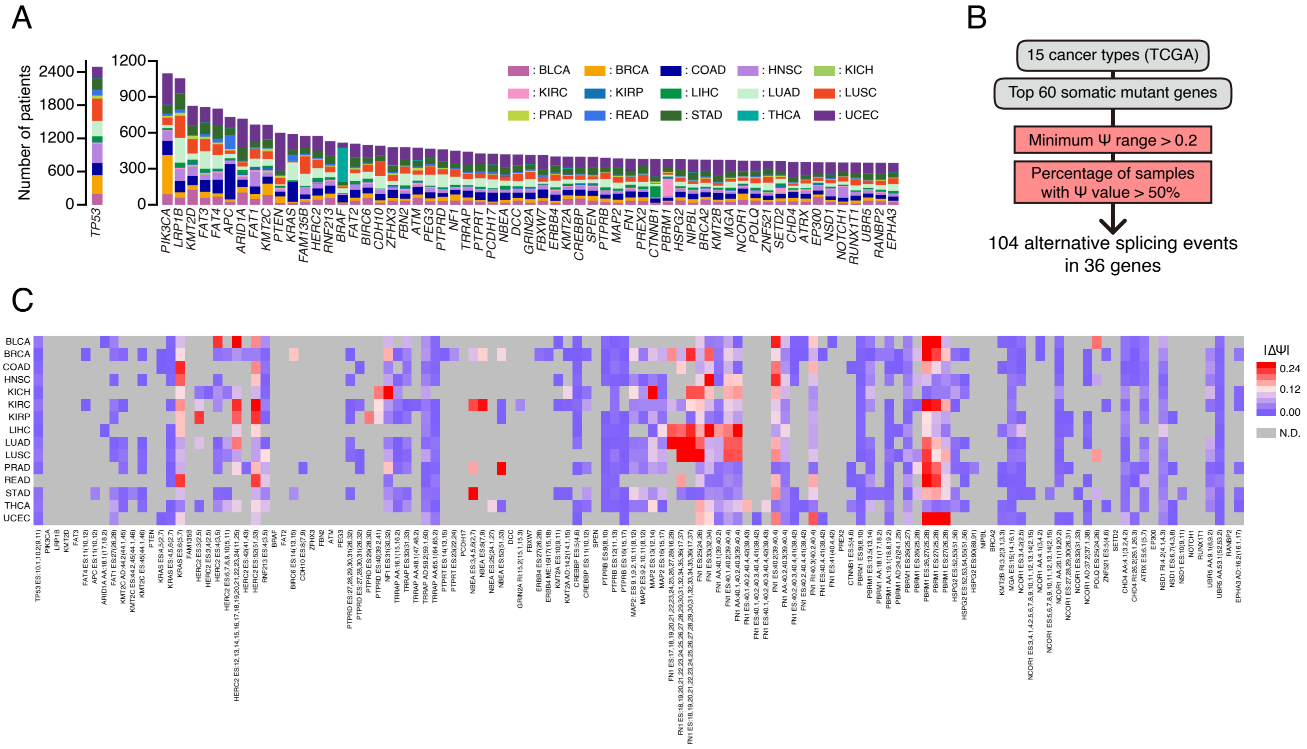

Appendix Figure S1. Comprehensive AS analysis in key cancer deriver genes.

(**A**) Bar graph displaying the top 60 mutated cancer driver genes in 15 cancer types. A list of candidate cancer driver genes was obtained from the Integrative OncoGenomics (IntOGen). (**B**) Flow chart showing the steps to identify AS events in the key cancer driver genes. (**C**) Heatmap showing changes in the mean PSI value in cancer tissues compared with that in normal tissues across AS events in the key cancer driver genes. Ψ, PSI. N.D., not detected.

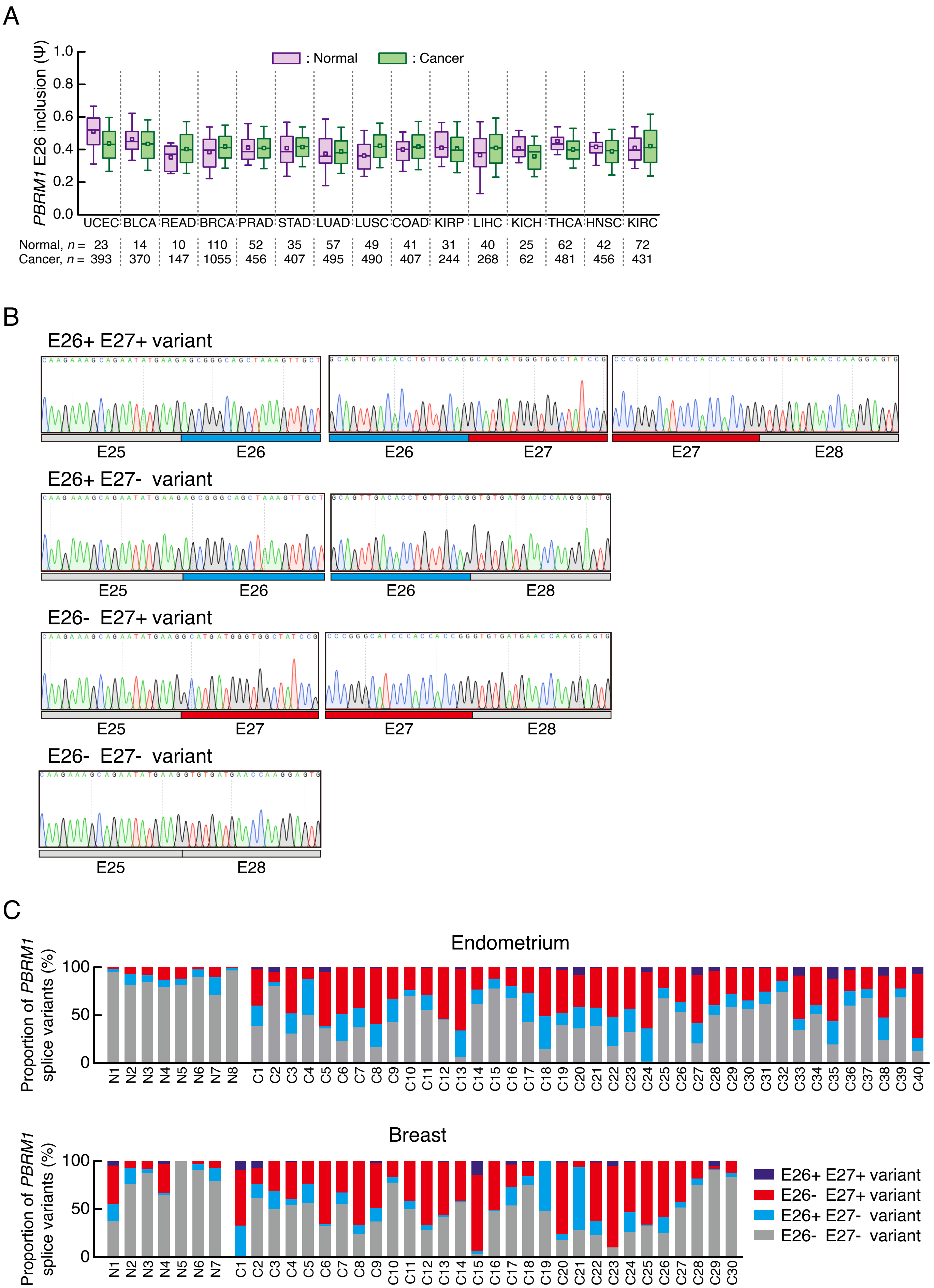
A
PBRM1 E26 inclusion (Ψ)
1.0
0.8
0.6
0.4
0.2
0.0
: Normal
: Cancer
UCEC BLCA READ BRCA PRAD STAD LUAD LUSC COAD KIRP LIHC KICH THCA HNSC KIRC
Normal, n = 23 14 10 110 52 35 57 49 41 31 40 25 62 42 72
Cancer, n = 393 370 147 1055 456 407 495 490 407 244 268 62 481 456 431
B
E26+ E27+ variant
E25 E26 E26 E27 E27 E28
E26+ E27- variant
E25 E26 E26 E28
E26- E27+ variant
E25 E27 E27 E28
E26- E27- variant
E25 E28
C
Endometrium
Proportion of PBRM1 splice variants (%)
100
50
0
N1 N2 N3 N4 N5 N6 N7 N8 C1 C2 C3 C4 C5 C6 C7 C8 C9 C10 C11 C12 C13 C14 C15 C16 C17 C18 C19 C20 C21 C22 C23 C24 C25 C26 C27 C28 C29 C30 C31 C32 C33 C34 C35 C36 C37 C38 C39 C40
Breast
Proportion of PBRM1 splice variants (%)
100
50
0
N1 N2 N3 N4 N5 N6 N7 C1 C2 C3 C4 C5 C6 C7 C8 C9 C10 C11 C12 C13 C14 C15 C16 C17 C18 C19 C20 C21 C22 C23 C24 C25 C26 C27 C28 C29 C30
E26+ E27+ variant
E26- E27+ variant
E26+ E27- variant
E26- E27- variant

Appendix Figure S2. AS pattern of *PBRM1* in cancer tissues.

(**A**) Box plots displaying PSI values of *PBRM1* E26 in TCGA database. n, number of samples; Ψ, PSI. Boxes represent the median, quartiles, 10th percentile, and 90th percentile. (**B**) Sanger sequencing analysis of the PCR products to confirm the *PBRM1* splice variants. Chromatogram shows the splice site positions between the indicated exons of *PBRM1*. (**C**) Proportion of *PBRM1* splice variants in cDNA arrays of endometrium (top) and breast (bottom) samples, including cancer (C) and healthy (N) tissues. The bar graphs represent the ratios of each *PBRM1* splice variant.

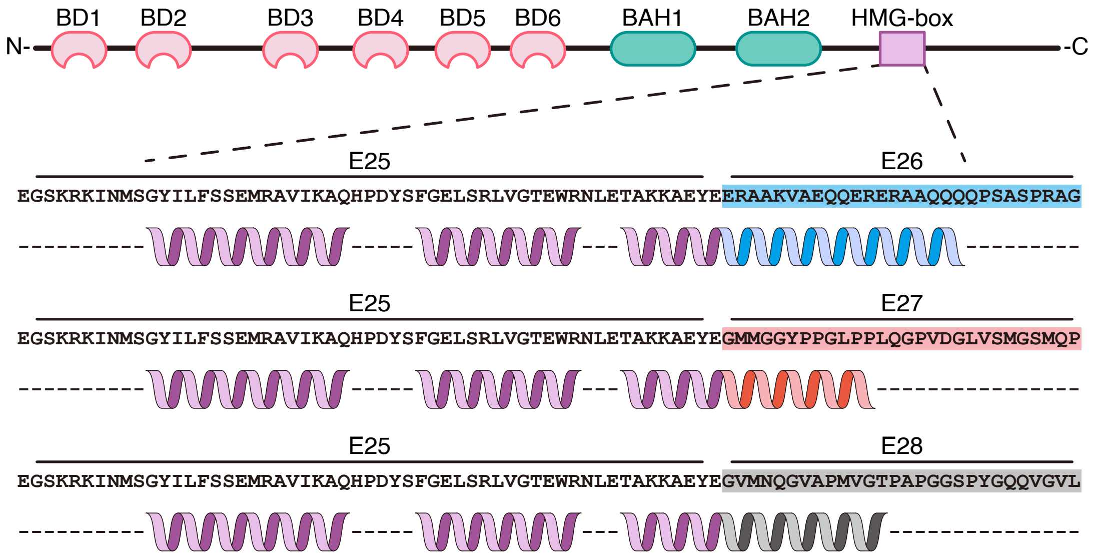

Appendix Figure S3. Predicted secondary structure of the E27 region in PBRM1 splicing isoforms.

Diagrammatic representing *in silico* secondary structures of HMG-box domain within PBRM1 splicing isoforms. Jpred4-predicted α-helical structures are shown at the bottom of the amino acid sequences of each PBRM1 splicing isoform. BD, bromodomain; BAH, bromodomain-adjacent homology; HMG-box, high mobility group box.
