# Supplementary figures and images for: Alternative splicing of PBRM1 mediates resistance to PD-1 blockade therapy in renal cancer
Source: EMBO J. 2024 Oct 7;43(22):7. doi: 10.1038/s44318-024-00262-7 (PMC11574163; doi:10.1038/s44318-024-00262-7)

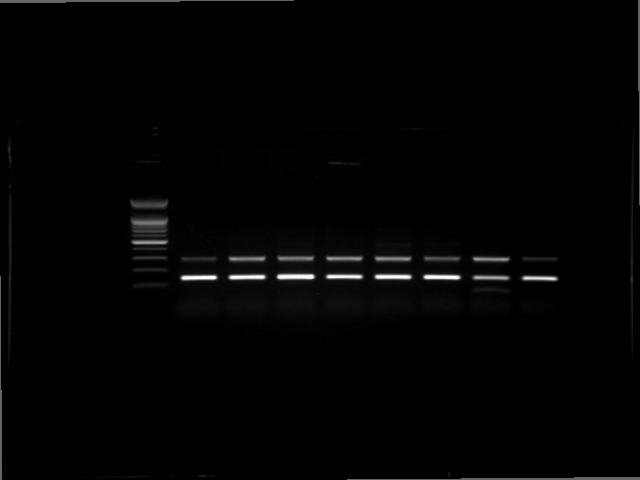

Supplement: Supplementary file 16 — Source data Fig. 1 [file 44318_2024_262_MOESM16_ESM.zip › Figure 1/1E/RT-PCR-1.tif]

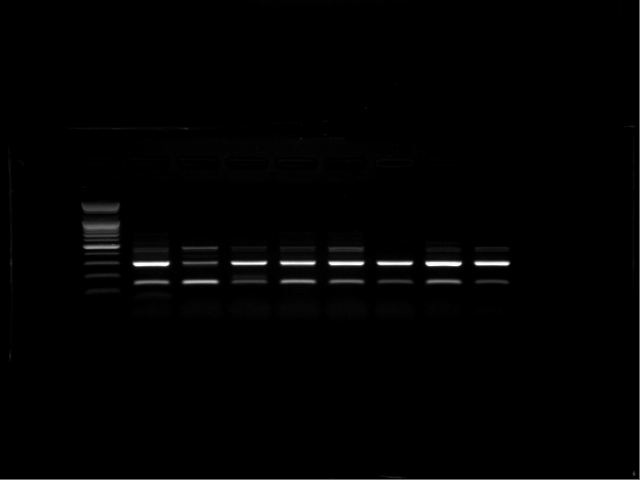

Supplement: Supplementary file 16 — Source data Fig. 1 [file 44318_2024_262_MOESM16_ESM.zip › Figure 1/1E/RT-PCR-2.tif]

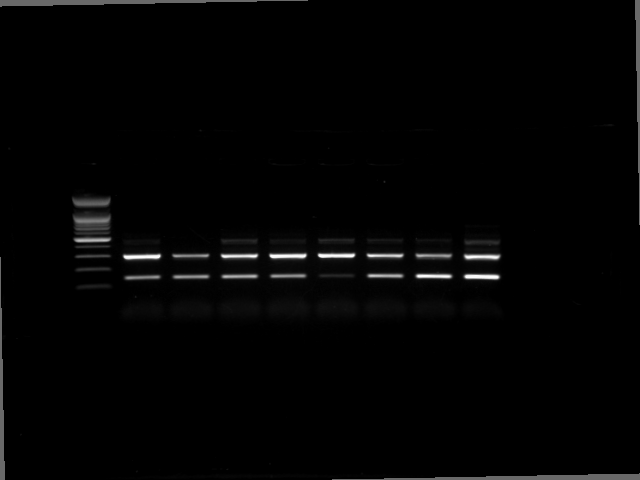

Supplement: Supplementary file 16 — Source data Fig. 1 [file 44318_2024_262_MOESM16_ESM.zip › Figure 1/1E/RT-PCR-3.tif]

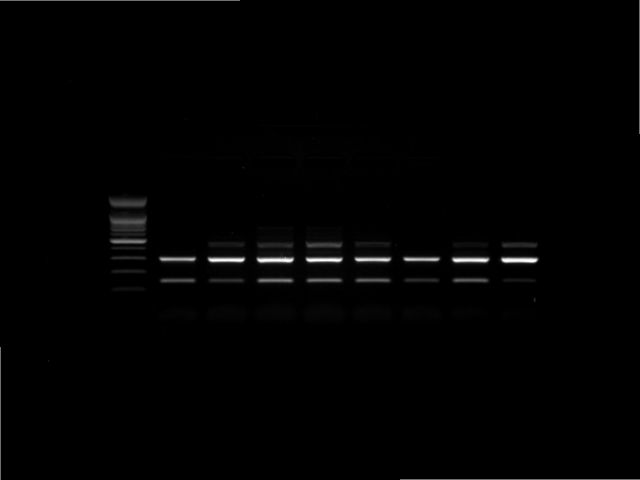

Supplement: Supplementary file 16 — Source data Fig. 1 [file 44318_2024_262_MOESM16_ESM.zip › Figure 1/1E/RT-PCR-4.tif]

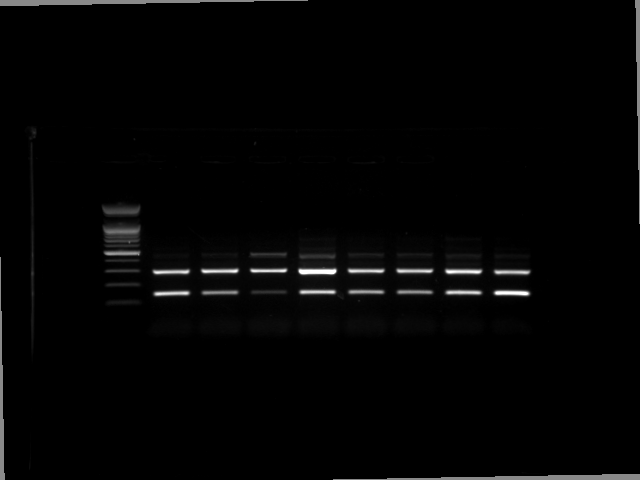

Supplement: Supplementary file 16 — Source data Fig. 1 [file 44318_2024_262_MOESM16_ESM.zip › Figure 1/1E/RT-PCR-5.tif]

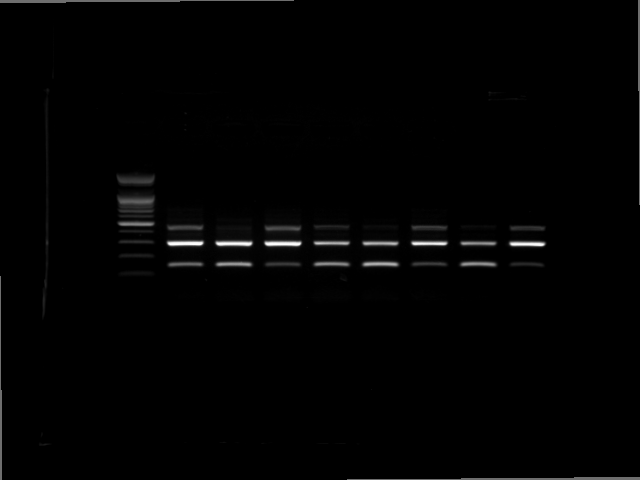

Supplement: Supplementary file 16 — Source data Fig. 1 [file 44318_2024_262_MOESM16_ESM.zip › Figure 1/1E/RT-PCR-6.tif]

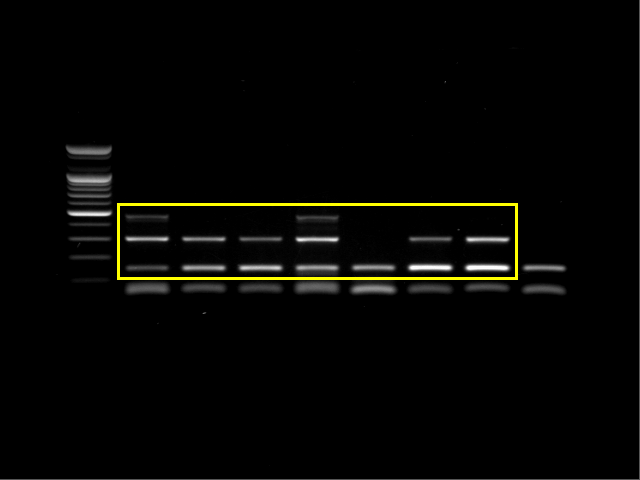

Supplement: Supplementary file 16 — Source data Fig. 1 [file 44318_2024_262_MOESM16_ESM.zip › Figure 1/1F/RT-PCR-1.tif]

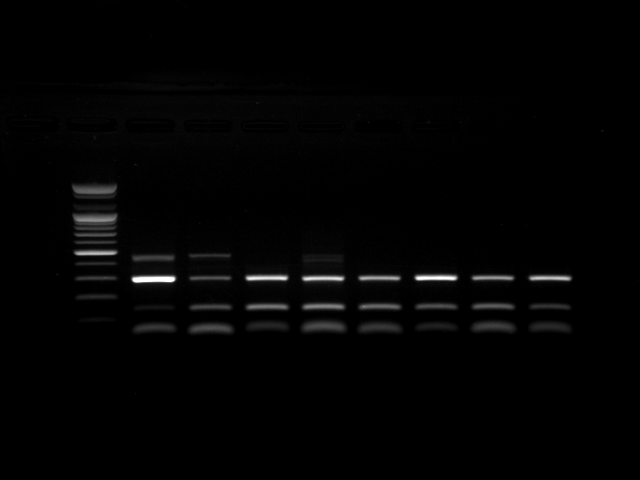

Supplement: Supplementary file 16 — Source data Fig. 1 [file 44318_2024_262_MOESM16_ESM.zip › Figure 1/1F/RT-PCR-2.tif]

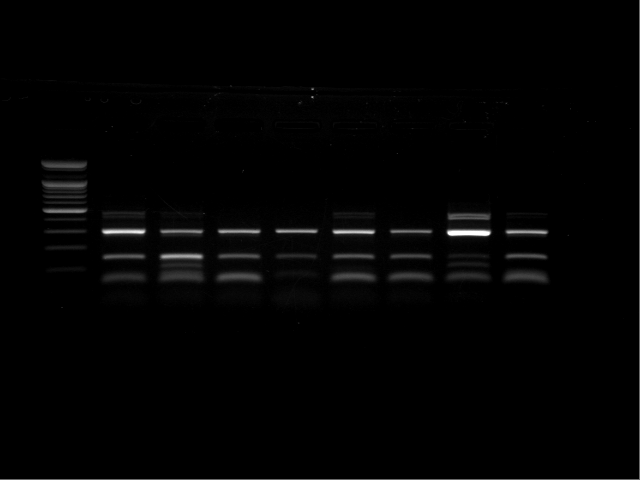

Supplement: Supplementary file 16 — Source data Fig. 1 [file 44318_2024_262_MOESM16_ESM.zip › Figure 1/1F/RT-PCR-3.tif]

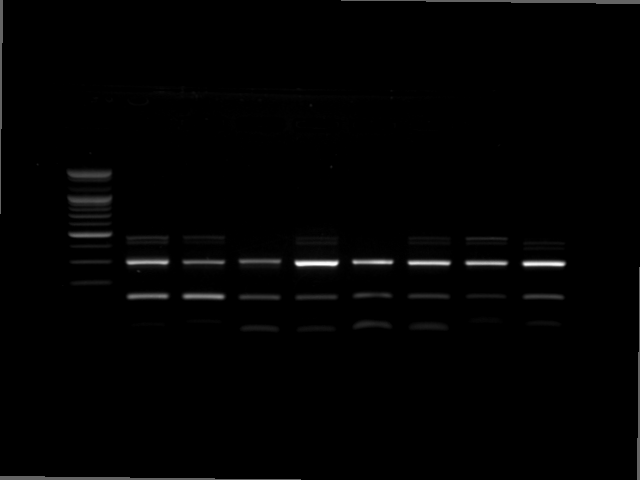

Supplement: Supplementary file 16 — Source data Fig. 1 [file 44318_2024_262_MOESM16_ESM.zip › Figure 1/1F/RT-PCR-4.tif]

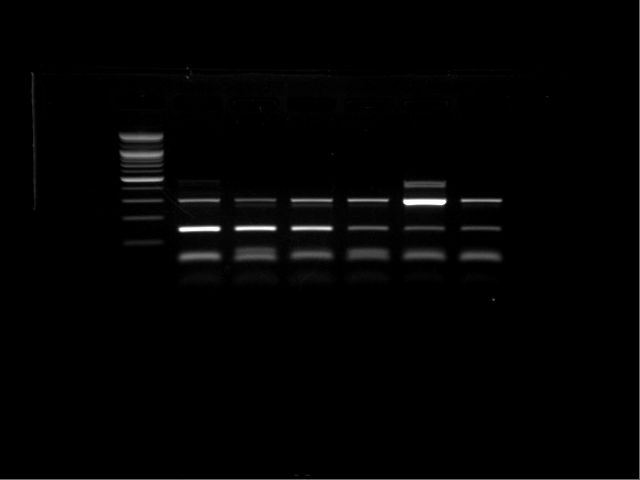

Supplement: Supplementary file 16 — Source data Fig. 1 [file 44318_2024_262_MOESM16_ESM.zip › Figure 1/1F/RT-PCR-5.tif]

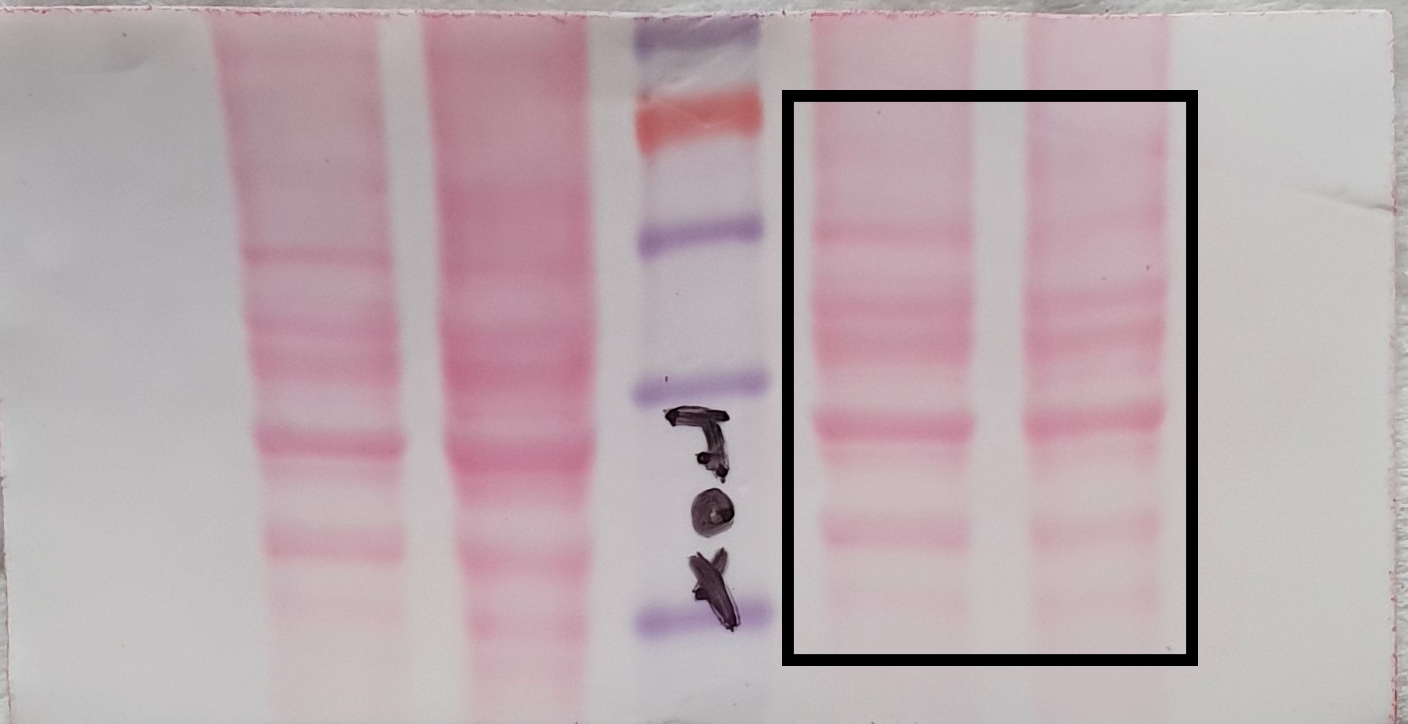

Supplement: Supplementary file 17 — Source data Fig. 2 [file 44318_2024_262_MOESM17_ESM.zip › Figure 2/2E/Ponceau S.tif]

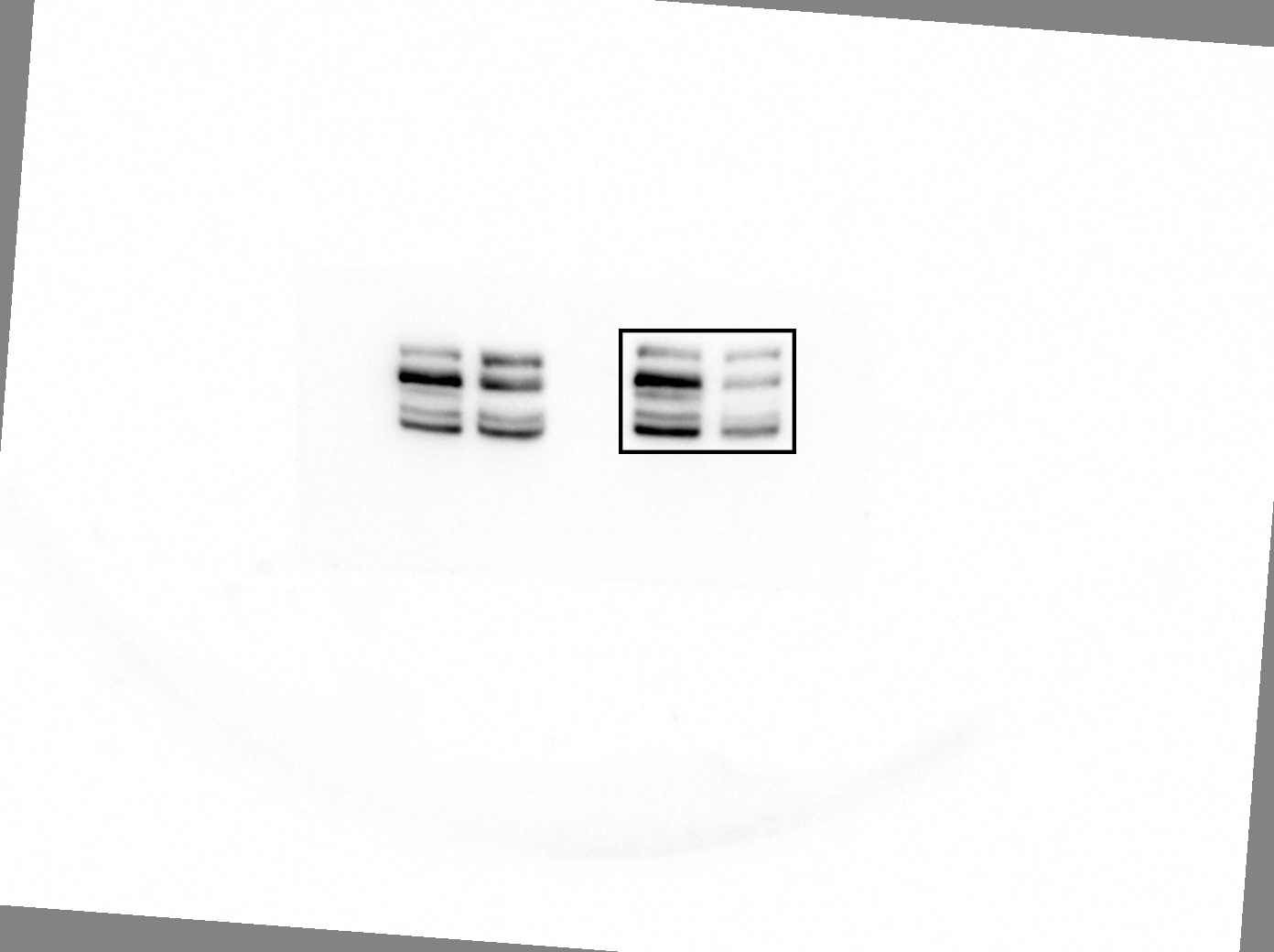

Supplement: Supplementary file 17 — Source data Fig. 2 [file 44318_2024_262_MOESM17_ESM.zip › Figure 2/2E/RBFOX2.tif]

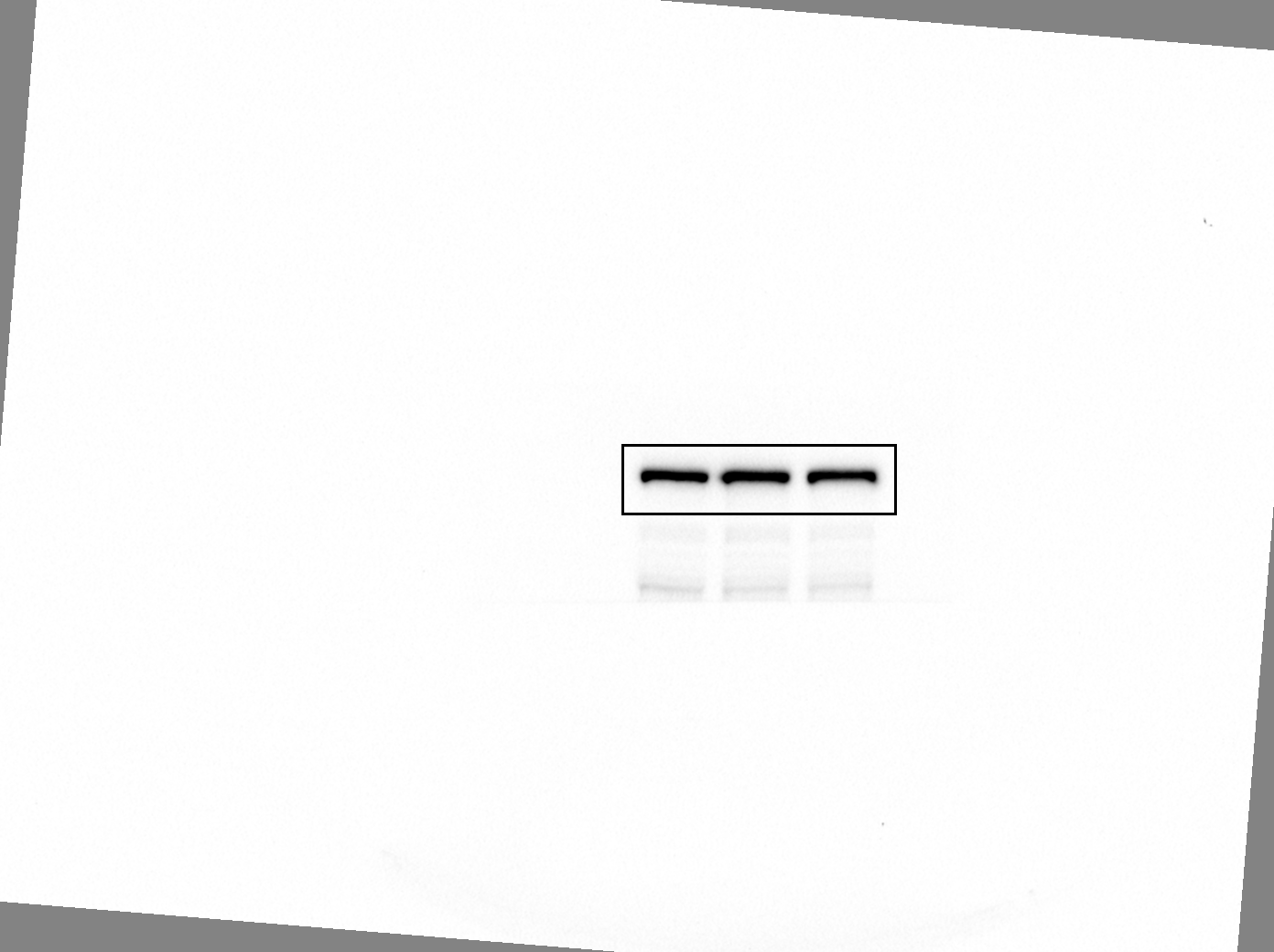

Supplement: Supplementary file 17 — Source data Fig. 2 [file 44318_2024_262_MOESM17_ESM.zip › Figure 2/2F/IB-PBRM1.tif]

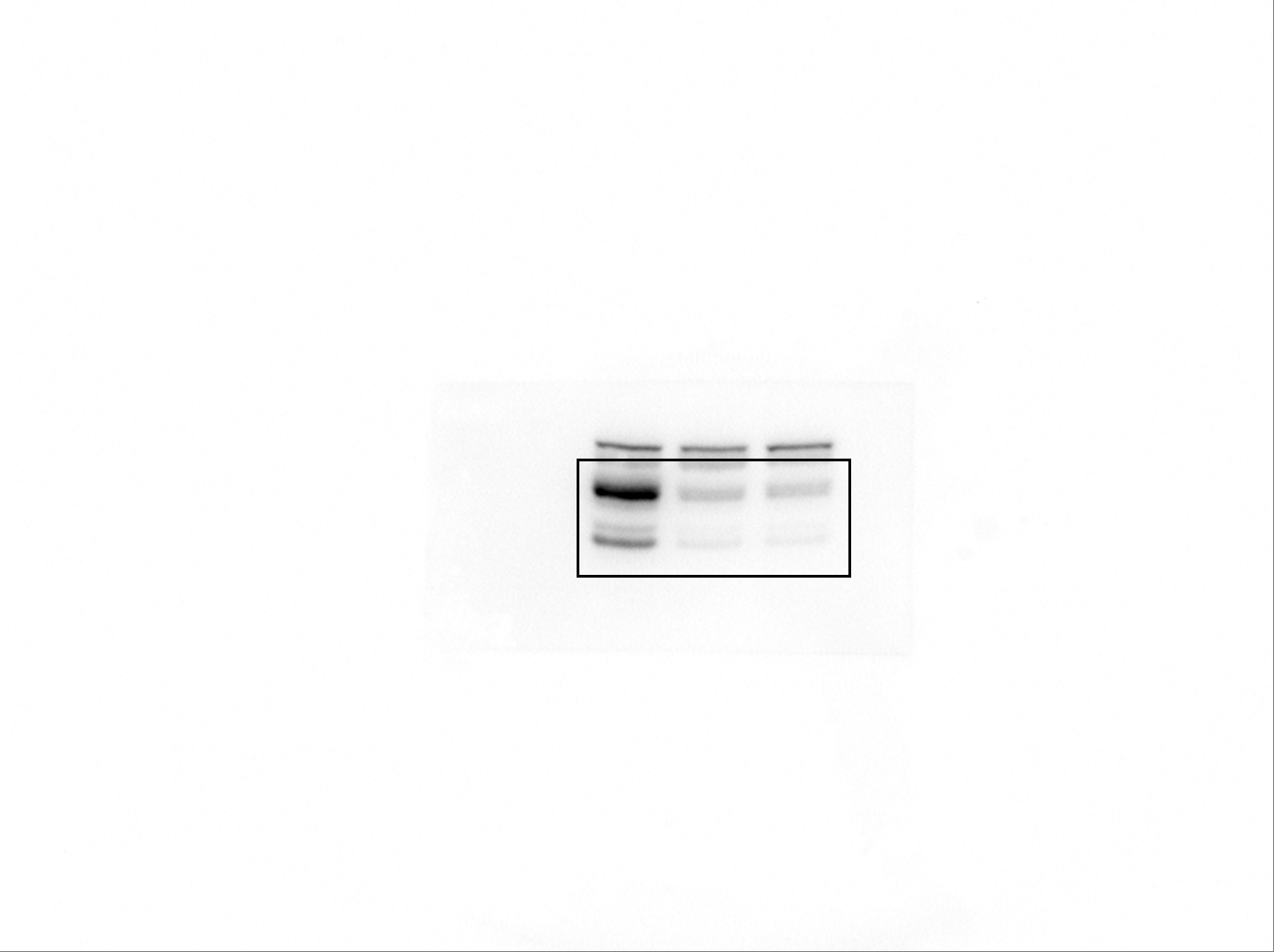

Supplement: Supplementary file 17 — Source data Fig. 2 [file 44318_2024_262_MOESM17_ESM.zip › Figure 2/2F/IB-RBFOX2.tif]

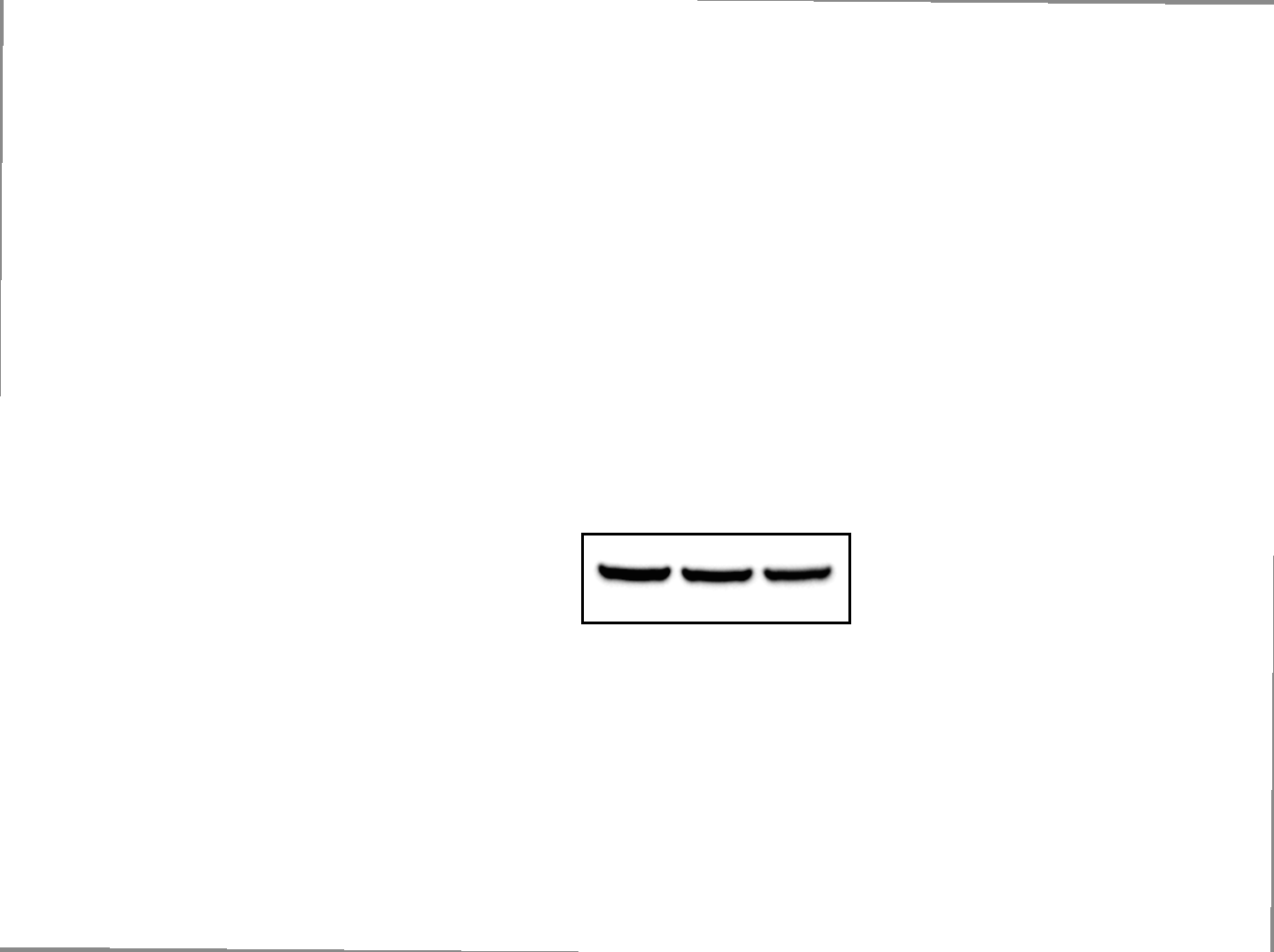

Supplement: Supplementary file 17 — Source data Fig. 2 [file 44318_2024_262_MOESM17_ESM.zip › Figure 2/2F/IB-Tubulin.tif]

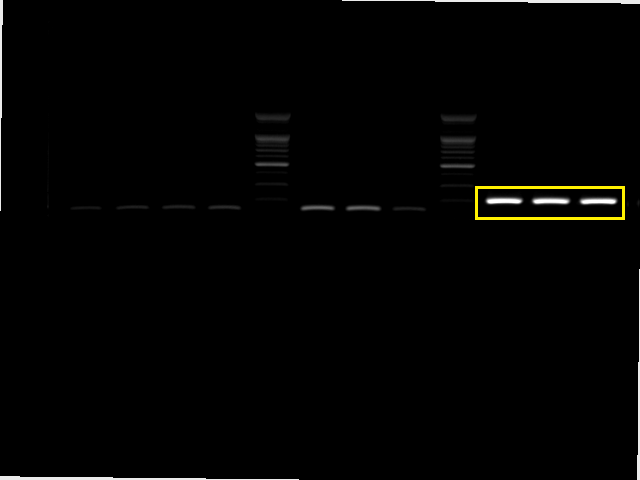

Supplement: Supplementary file 17 — Source data Fig. 2 [file 44318_2024_262_MOESM17_ESM.zip › Figure 2/2F/PCR-E22-E23.tif]

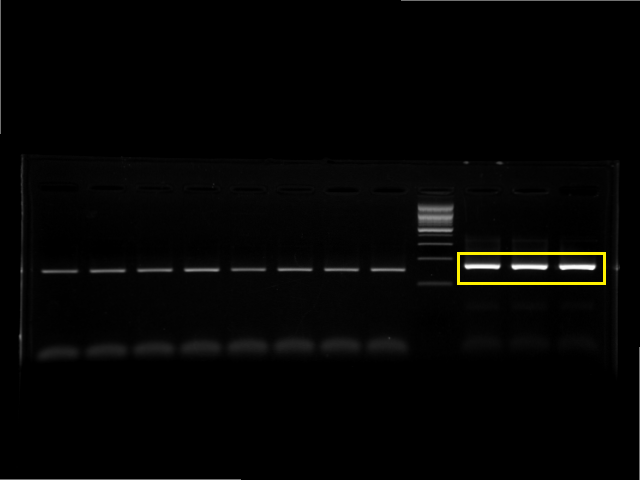

Supplement: Supplementary file 17 — Source data Fig. 2 [file 44318_2024_262_MOESM17_ESM.zip › Figure 2/2F/PCR-E25-E26.tif]

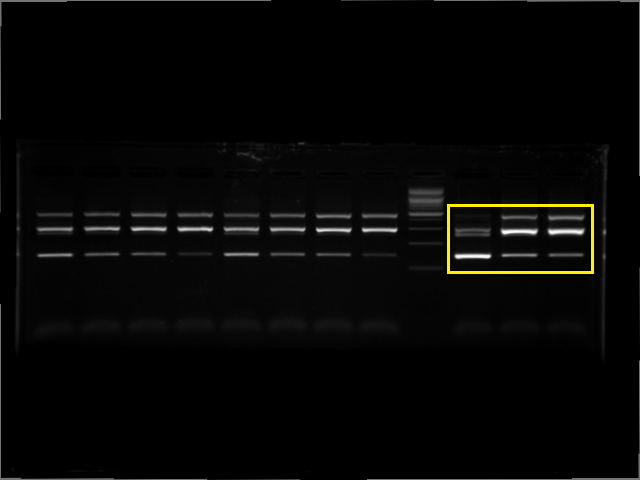

Supplement: Supplementary file 17 — Source data Fig. 2 [file 44318_2024_262_MOESM17_ESM.zip › Figure 2/2F/PCR-E25-E28.tif]

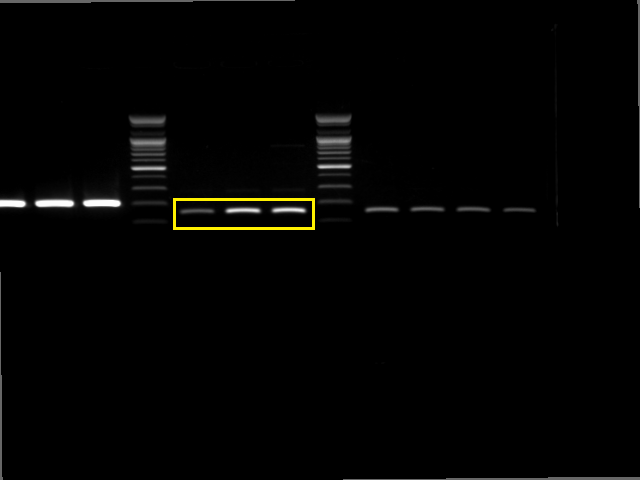

Supplement: Supplementary file 17 — Source data Fig. 2 [file 44318_2024_262_MOESM17_ESM.zip › Figure 2/2F/PCR-E27-E28.tif]

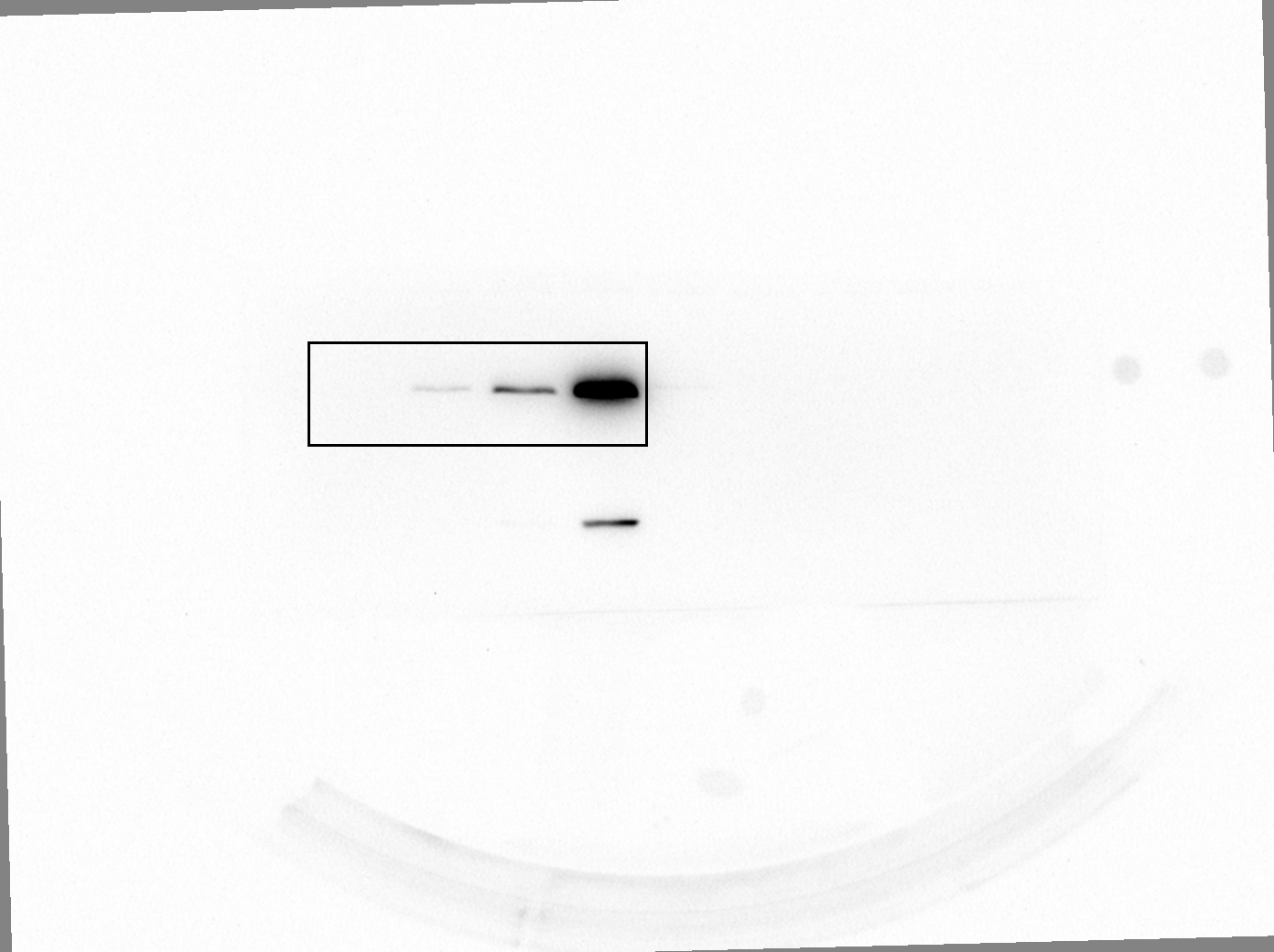

Supplement: Supplementary file 17 — Source data Fig. 2 [file 44318_2024_262_MOESM17_ESM.zip › Figure 2/2G/IB-FLAG.tif]

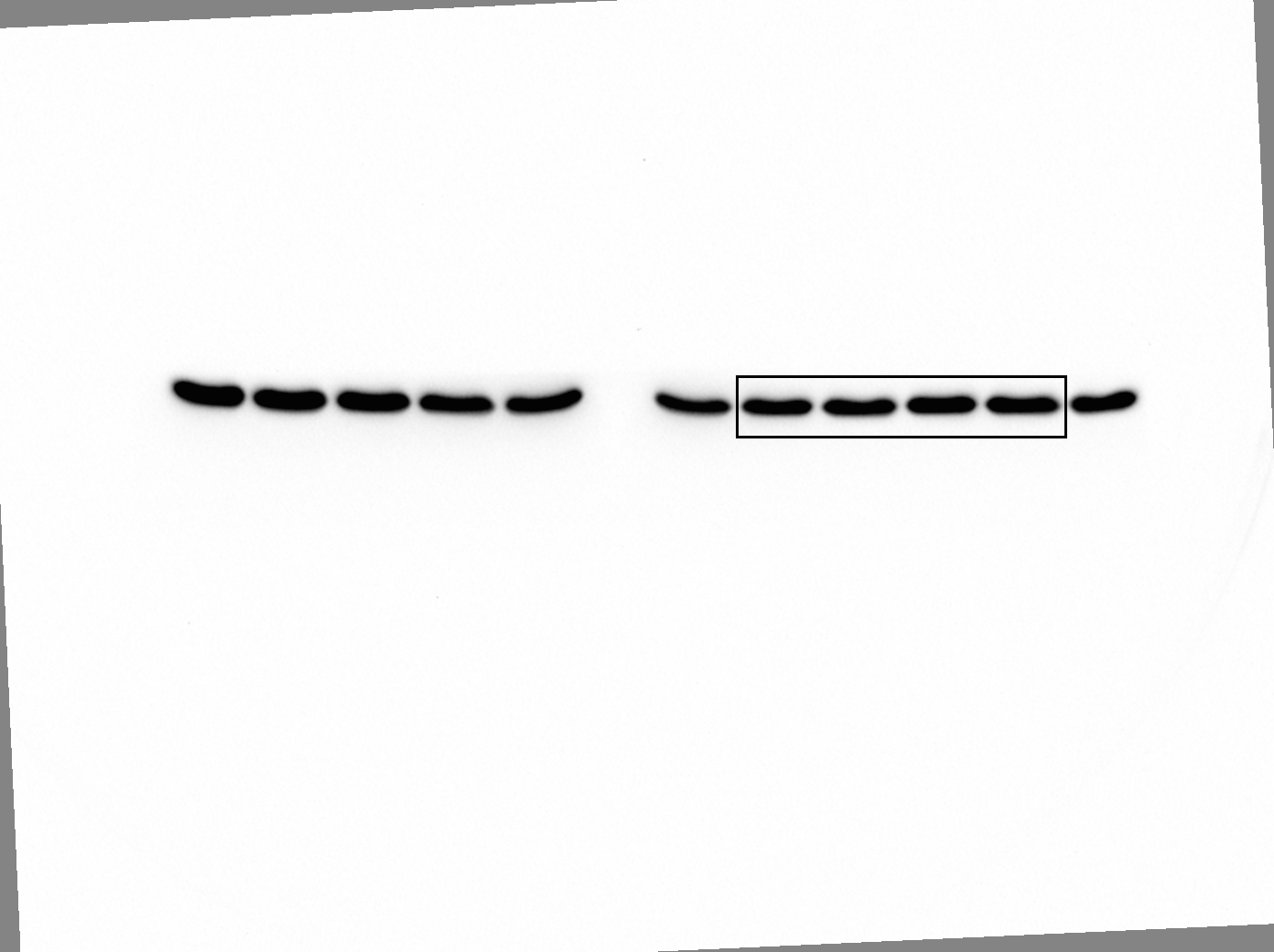

Supplement: Supplementary file 17 — Source data Fig. 2 [file 44318_2024_262_MOESM17_ESM.zip › Figure 2/2G/IB-GAPDH.tif]

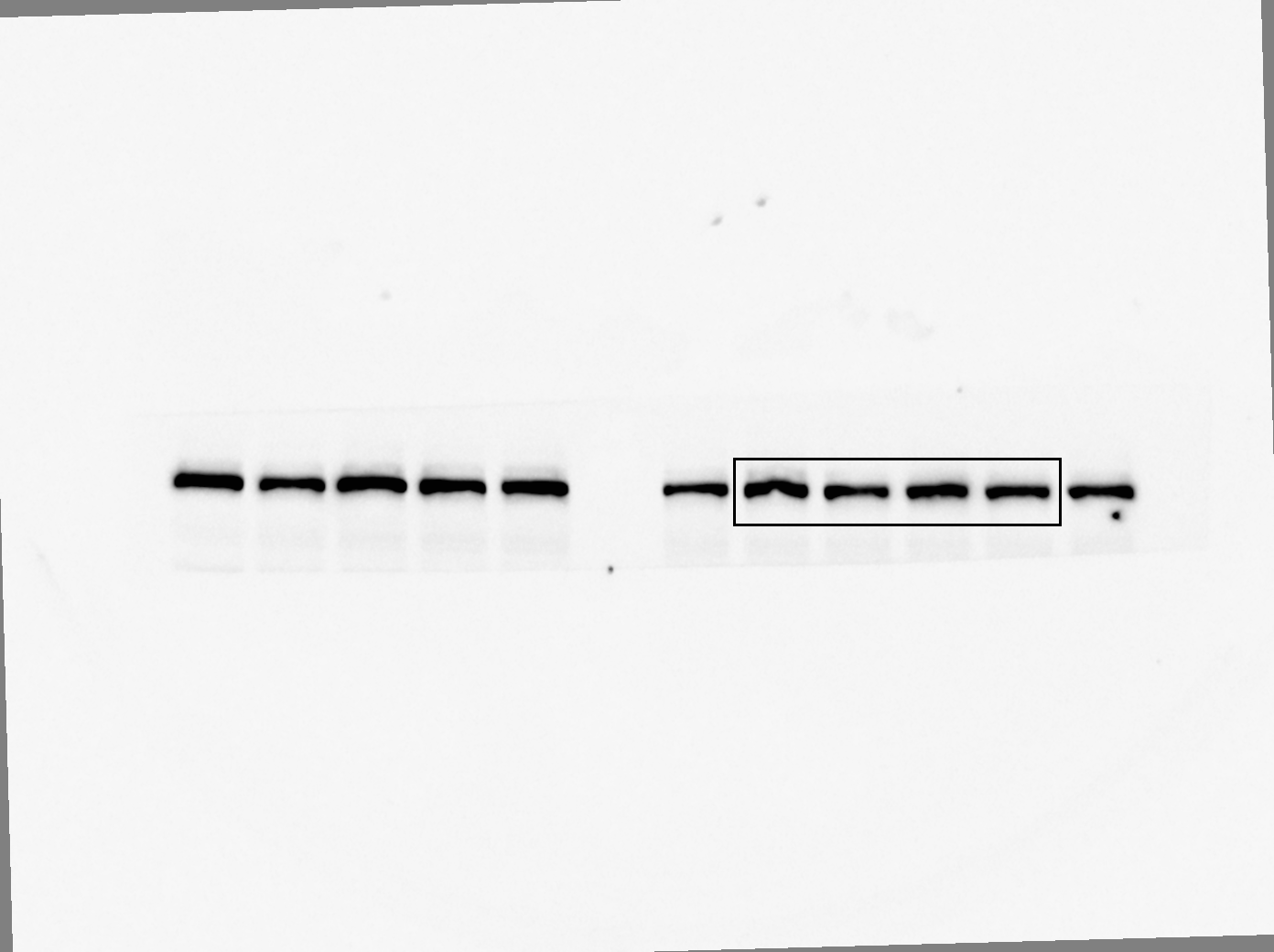

Supplement: Supplementary file 17 — Source data Fig. 2 [file 44318_2024_262_MOESM17_ESM.zip › Figure 2/2G/IB-PBRM1.tif]

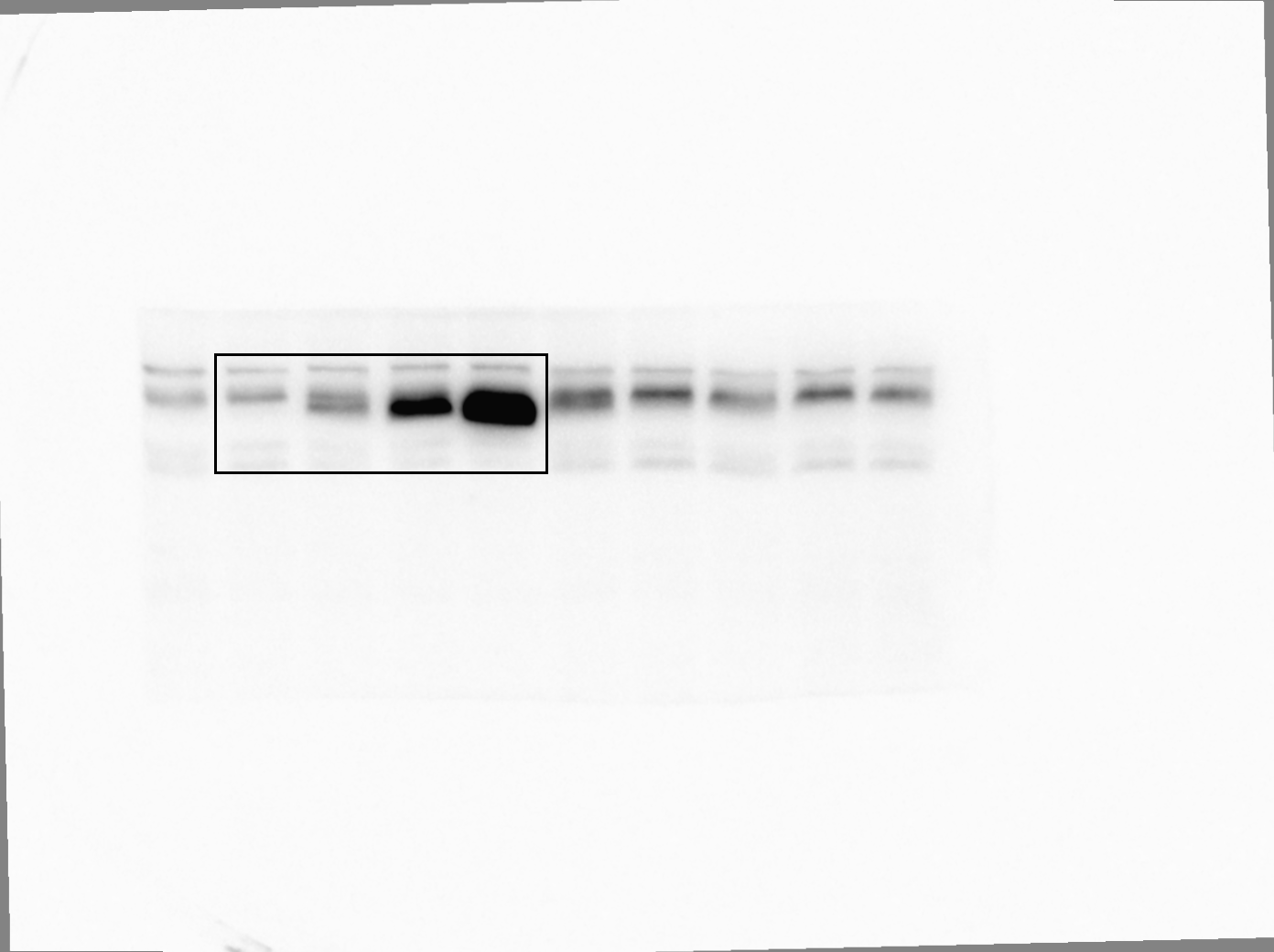

Supplement: Supplementary file 17 — Source data Fig. 2 [file 44318_2024_262_MOESM17_ESM.zip › Figure 2/2G/IB-RBFOX2.tif]

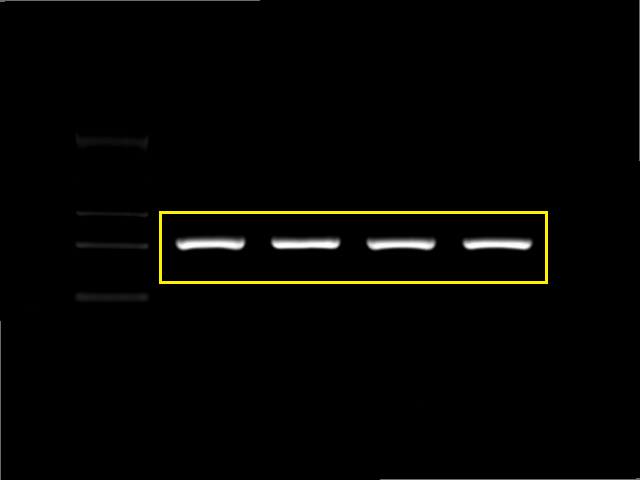

Supplement: Supplementary file 17 — Source data Fig. 2 [file 44318_2024_262_MOESM17_ESM.zip › Figure 2/2G/PCR-E22-E23.tif]

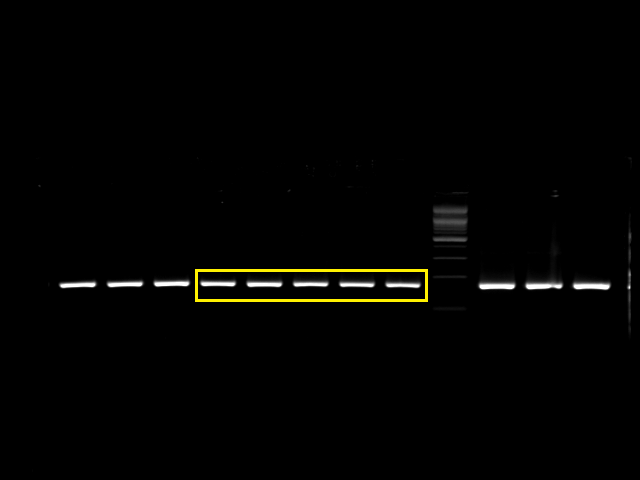

Supplement: Supplementary file 17 — Source data Fig. 2 [file 44318_2024_262_MOESM17_ESM.zip › Figure 2/2G/PCR-E25-E26.tif]

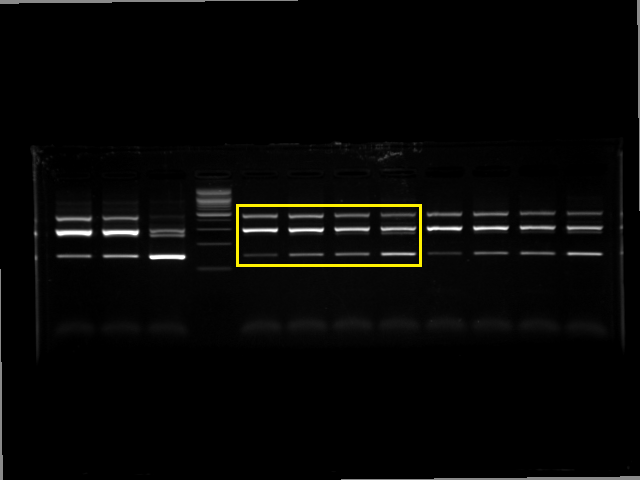

Supplement: Supplementary file 17 — Source data Fig. 2 [file 44318_2024_262_MOESM17_ESM.zip › Figure 2/2G/PCR-E25-E28.tif]

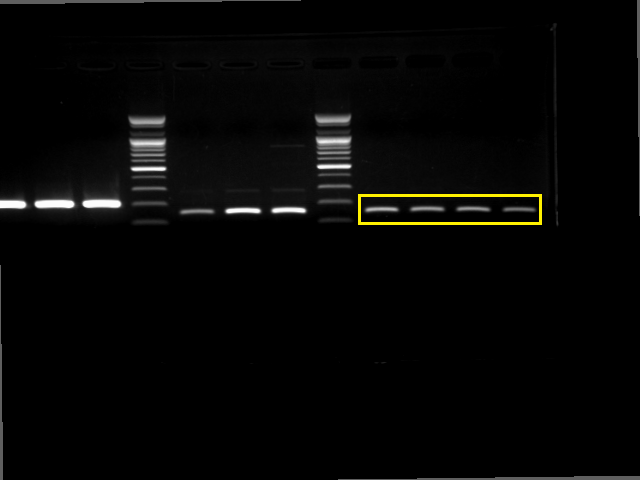

Supplement: Supplementary file 17 — Source data Fig. 2 [file 44318_2024_262_MOESM17_ESM.zip › Figure 2/2G/PCR-E27-E28.tif]

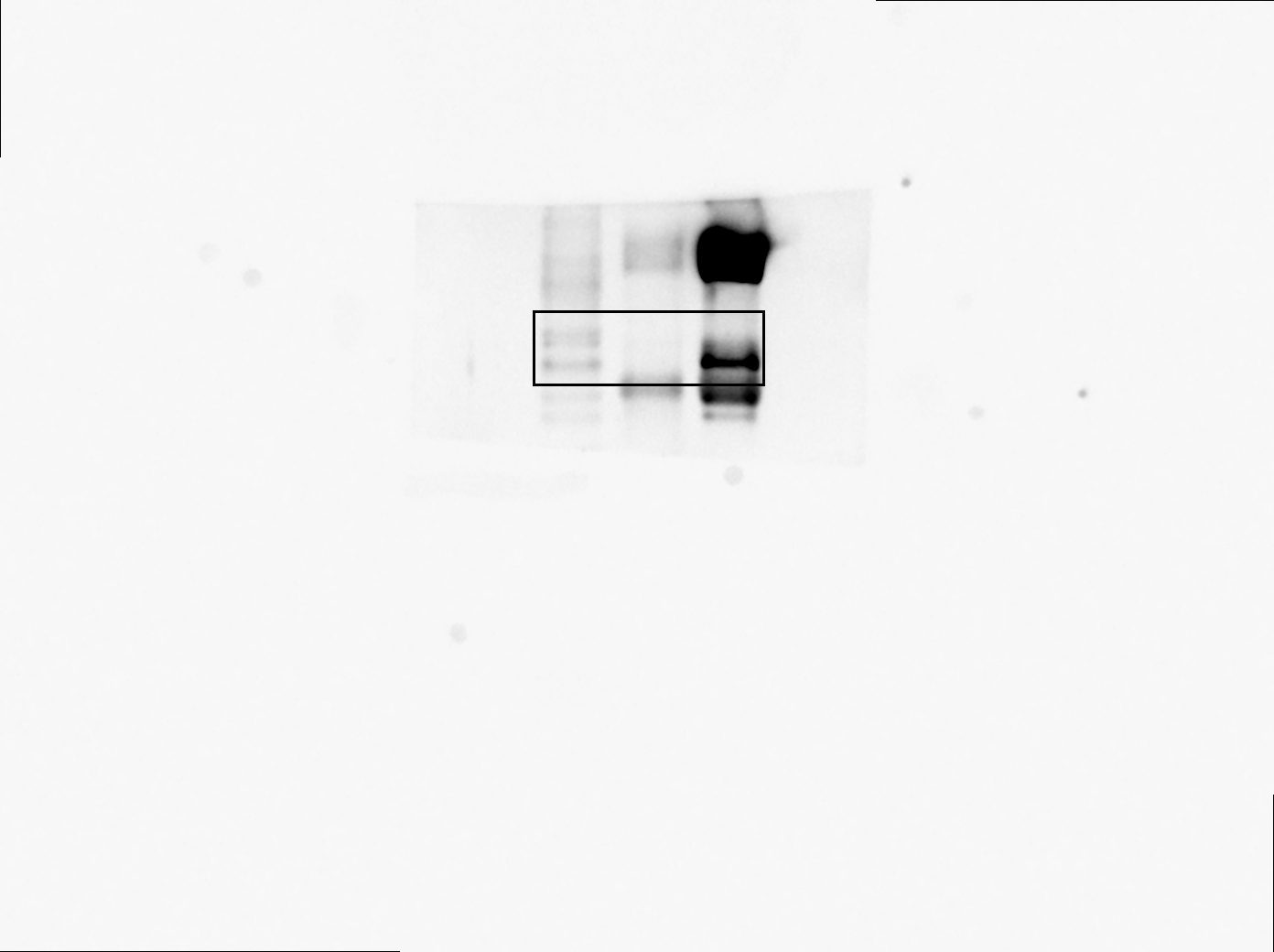

Supplement: Supplementary file 18 — Source data Fig. 3 [file 44318_2024_262_MOESM18_ESM.zip › Figure 3/3C/IB-RIP.tif]

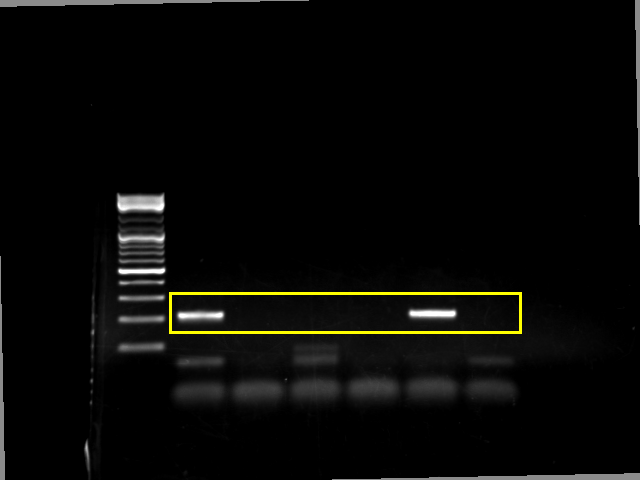

Supplement: Supplementary file 18 — Source data Fig. 3 [file 44318_2024_262_MOESM18_ESM.zip › Figure 3/3C/PCR-RIP.tif]

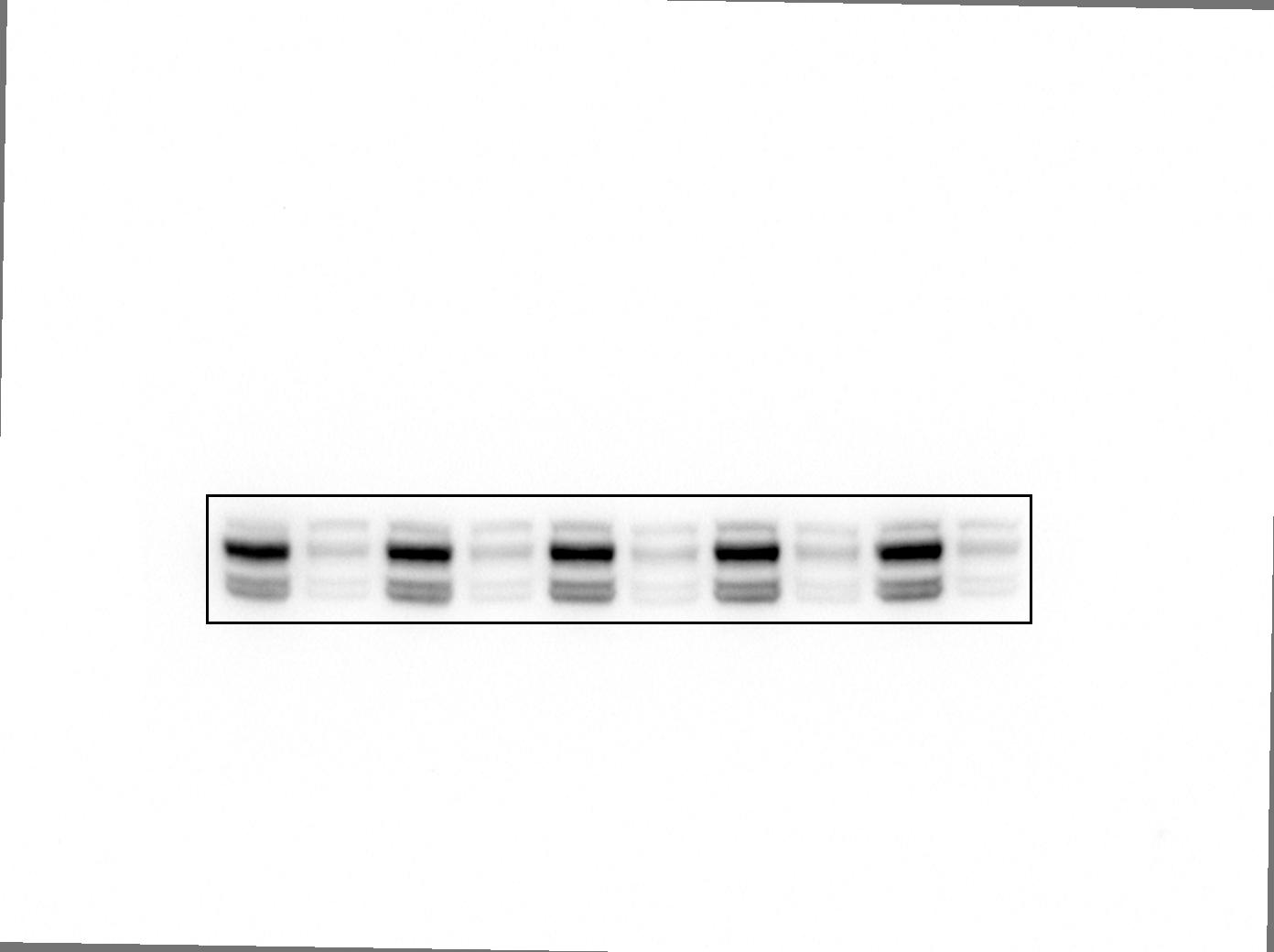

Supplement: Supplementary file 18 — Source data Fig. 3 [file 44318_2024_262_MOESM18_ESM.zip › Figure 3/3E/IB-RBFOX2.tif]

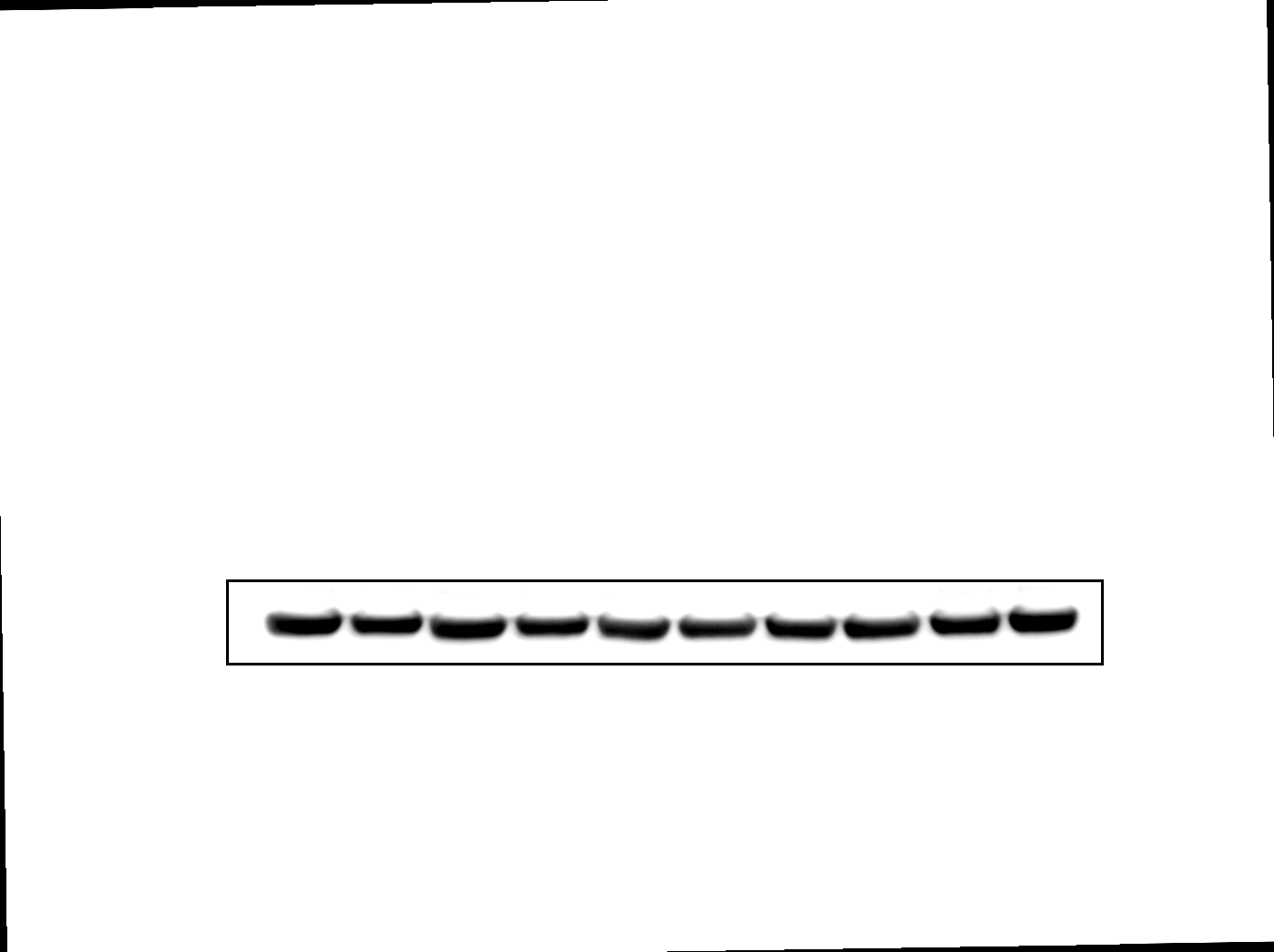

Supplement: Supplementary file 18 — Source data Fig. 3 [file 44318_2024_262_MOESM18_ESM.zip › Figure 3/3E/IP-GAPDH.tif]

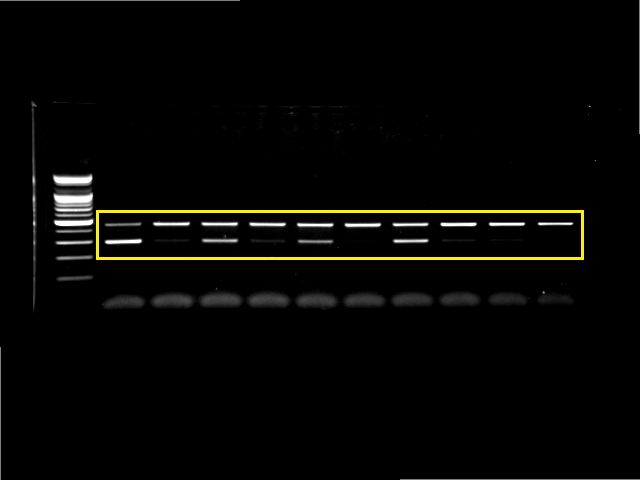

Supplement: Supplementary file 18 — Source data Fig. 3 [file 44318_2024_262_MOESM18_ESM.zip › Figure 3/3E/PCR-Mini.tif]

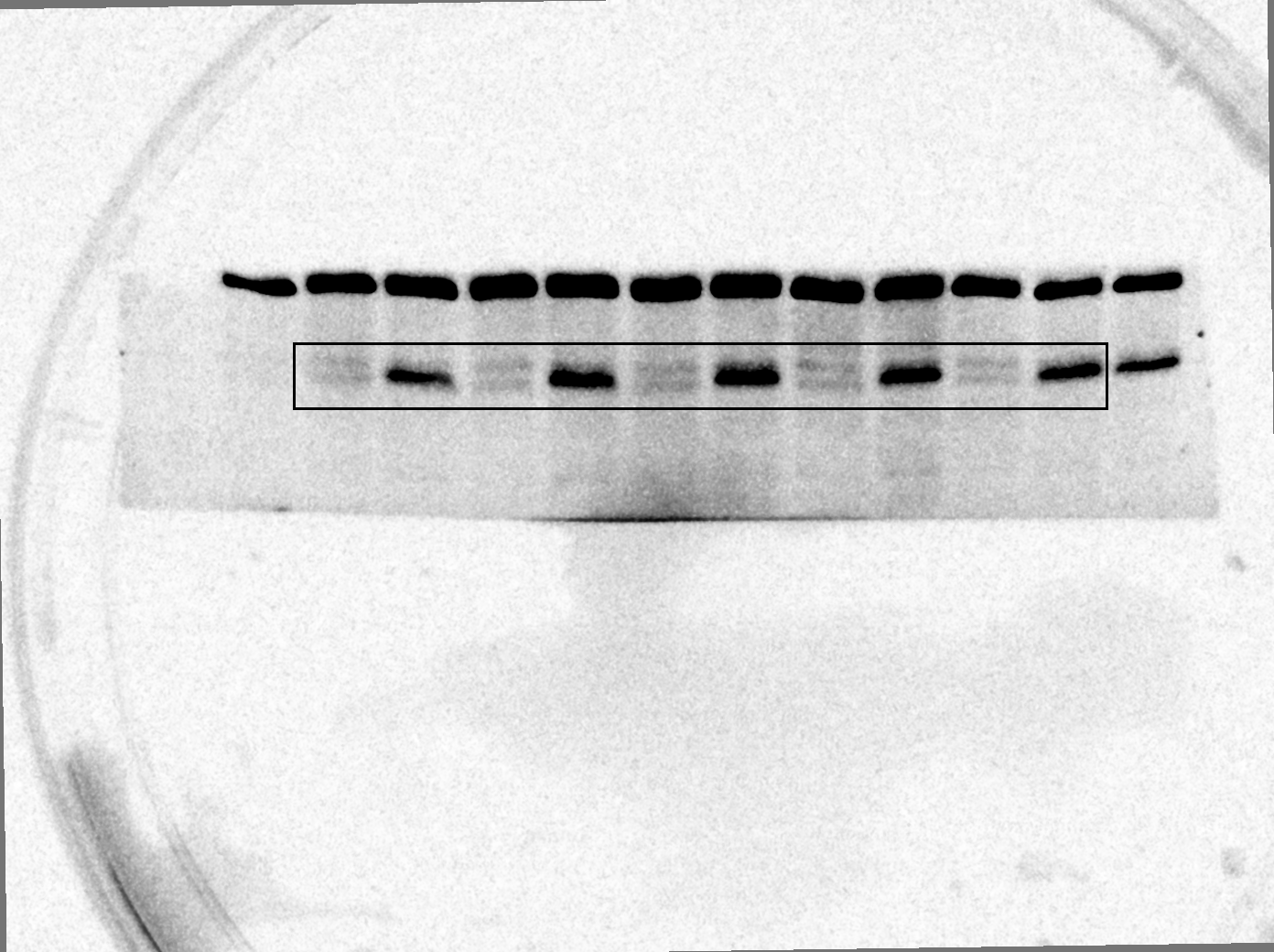

Supplement: Supplementary file 18 — Source data Fig. 3 [file 44318_2024_262_MOESM18_ESM.zip › Figure 3/3F/IP-FLAG.tif]

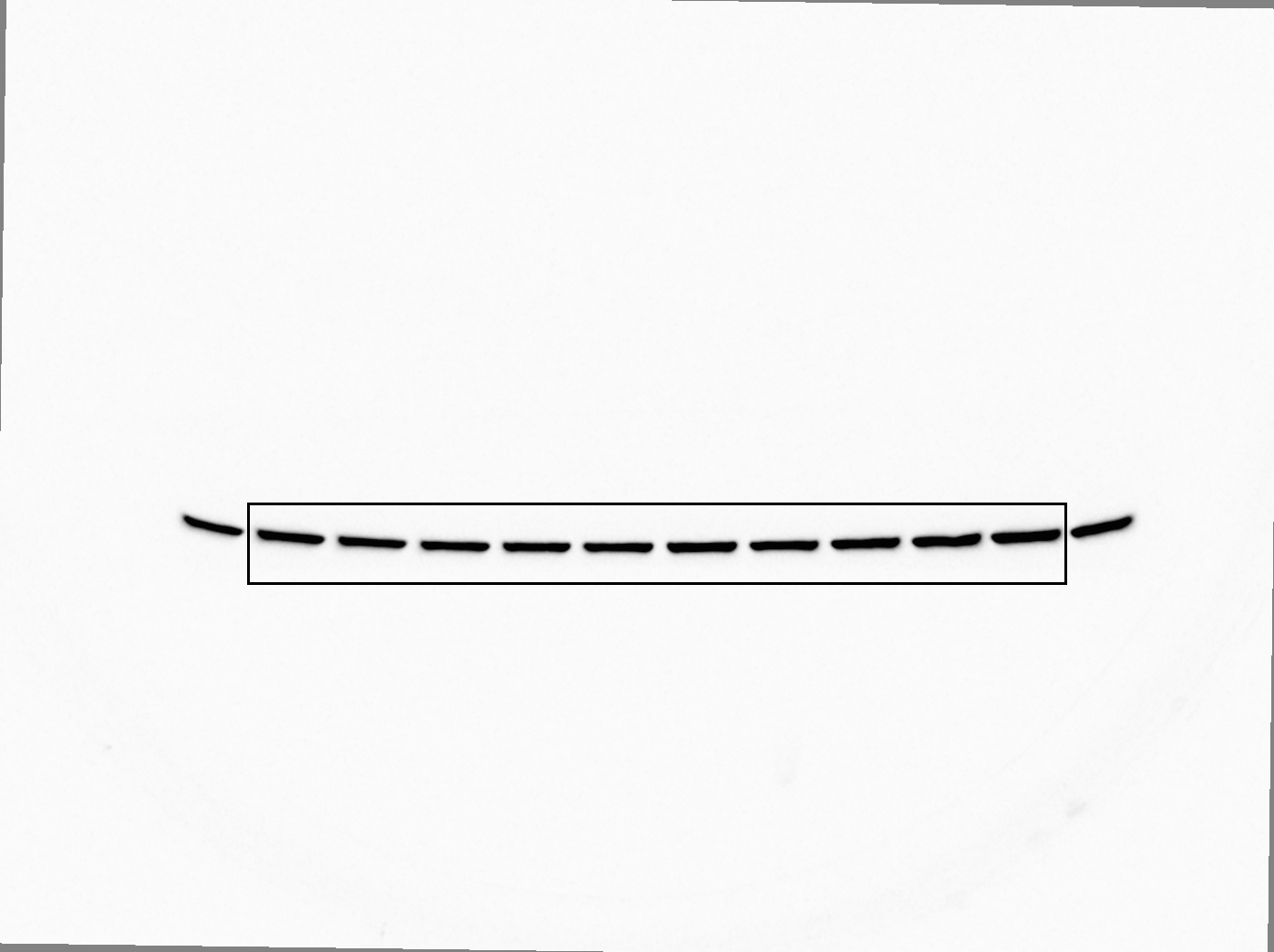

Supplement: Supplementary file 18 — Source data Fig. 3 [file 44318_2024_262_MOESM18_ESM.zip › Figure 3/3F/IP-Tubulin.tif]

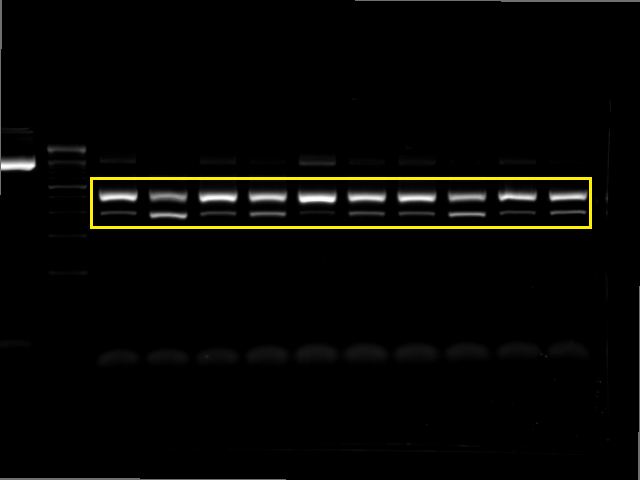

Supplement: Supplementary file 18 — Source data Fig. 3 [file 44318_2024_262_MOESM18_ESM.zip › Figure 3/3F/PCR-Mini.tif]

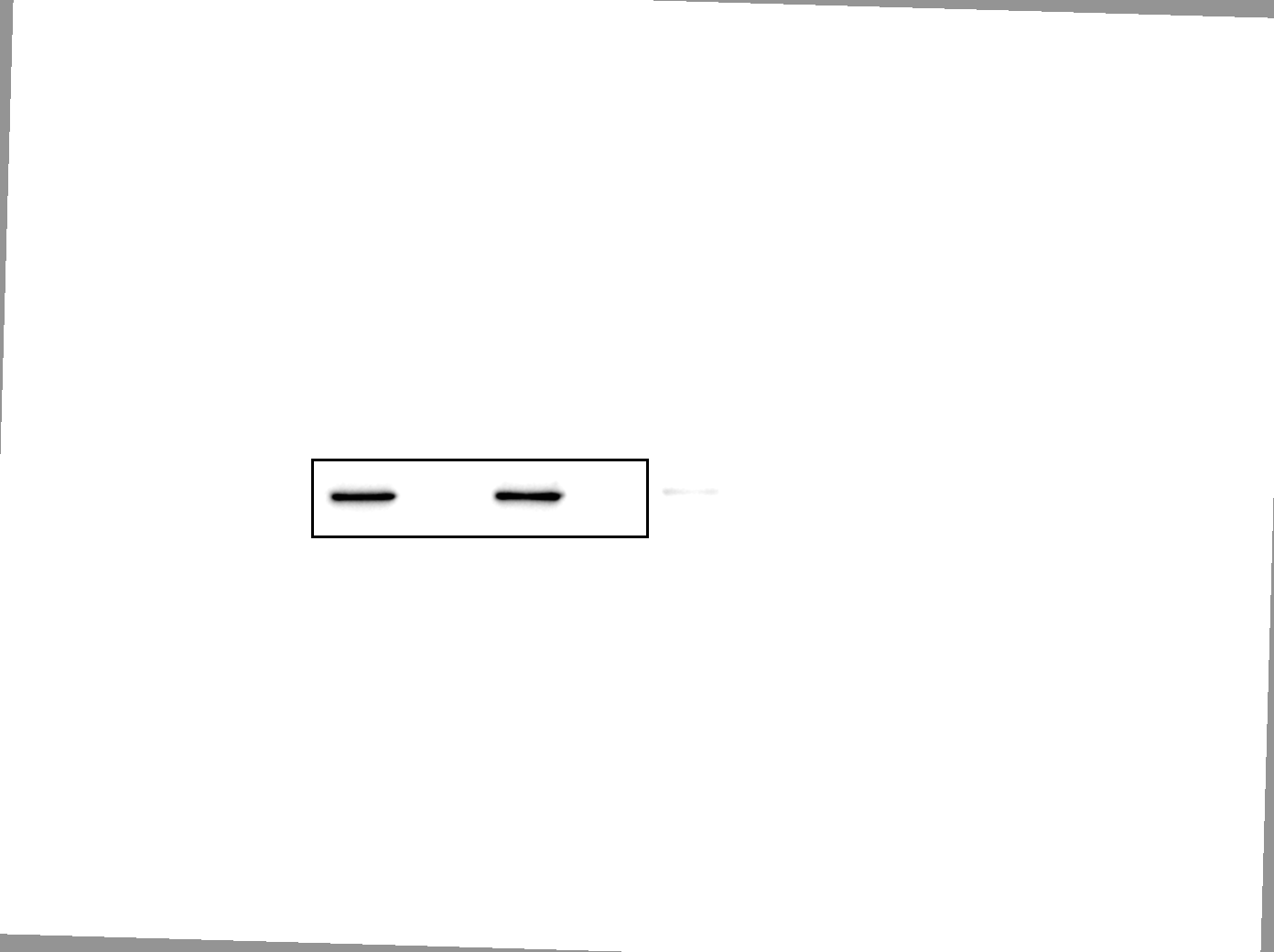

Supplement: Supplementary file 18 — Source data Fig. 3 [file 44318_2024_262_MOESM18_ESM.zip › Figure 3/3G/Input.tif]

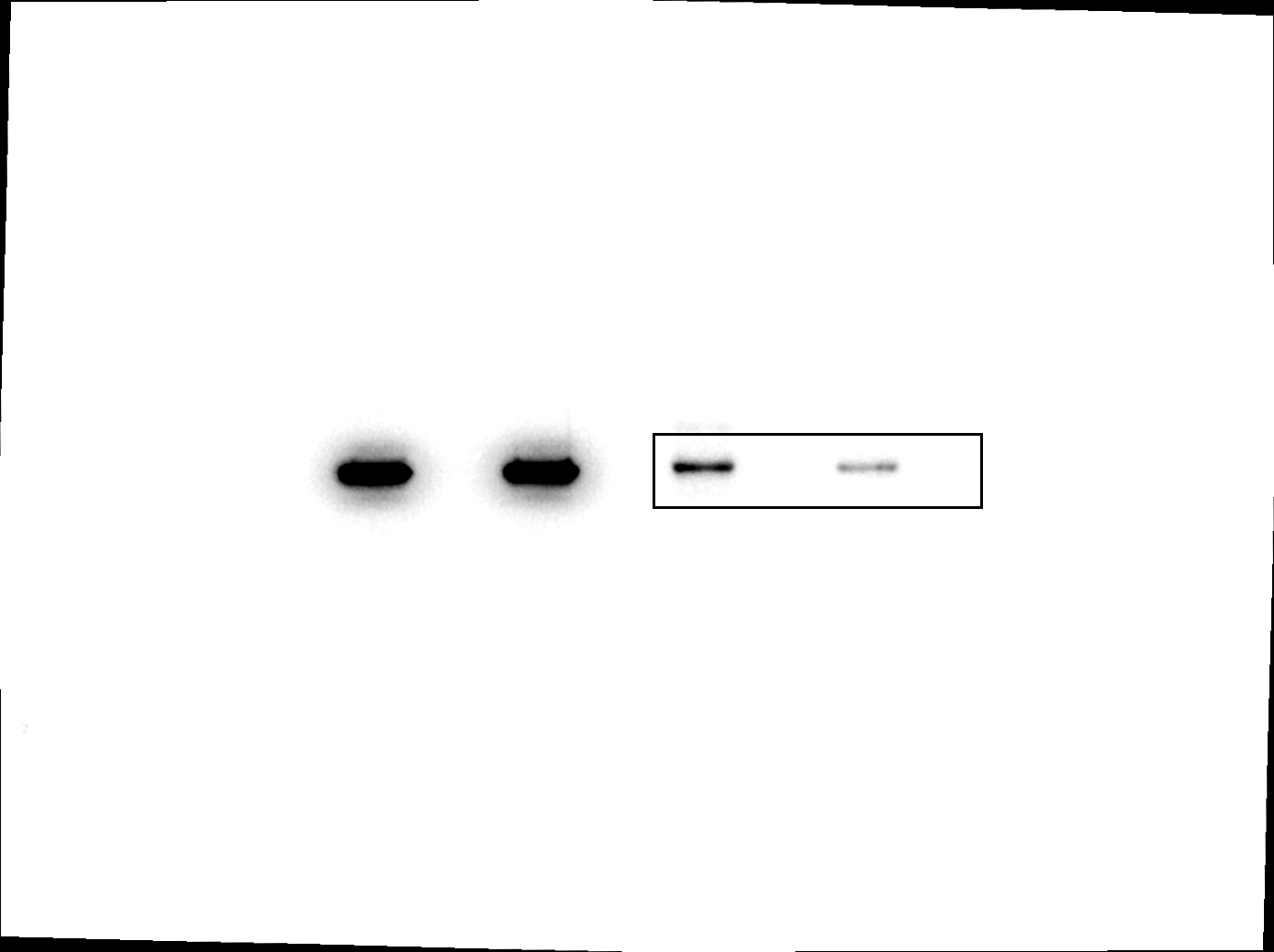

Supplement: Supplementary file 18 — Source data Fig. 3 [file 44318_2024_262_MOESM18_ESM.zip › Figure 3/3G/RNA pull-down.tif]

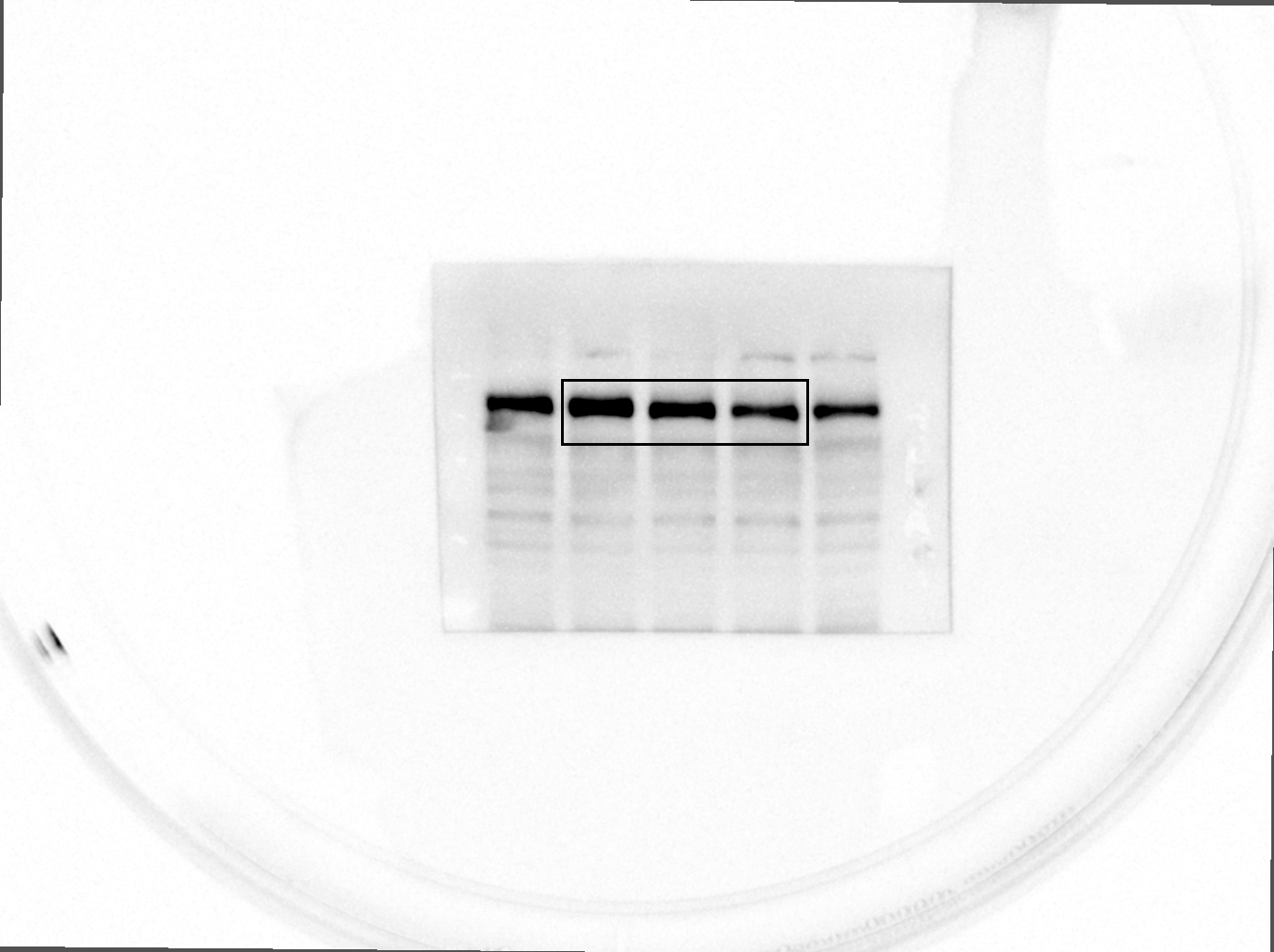

Supplement: Supplementary file 19 — Source data Fig. 4 [file 44318_2024_262_MOESM19_ESM.zip › Figure 4/4A/IB-PBRM1.tif]

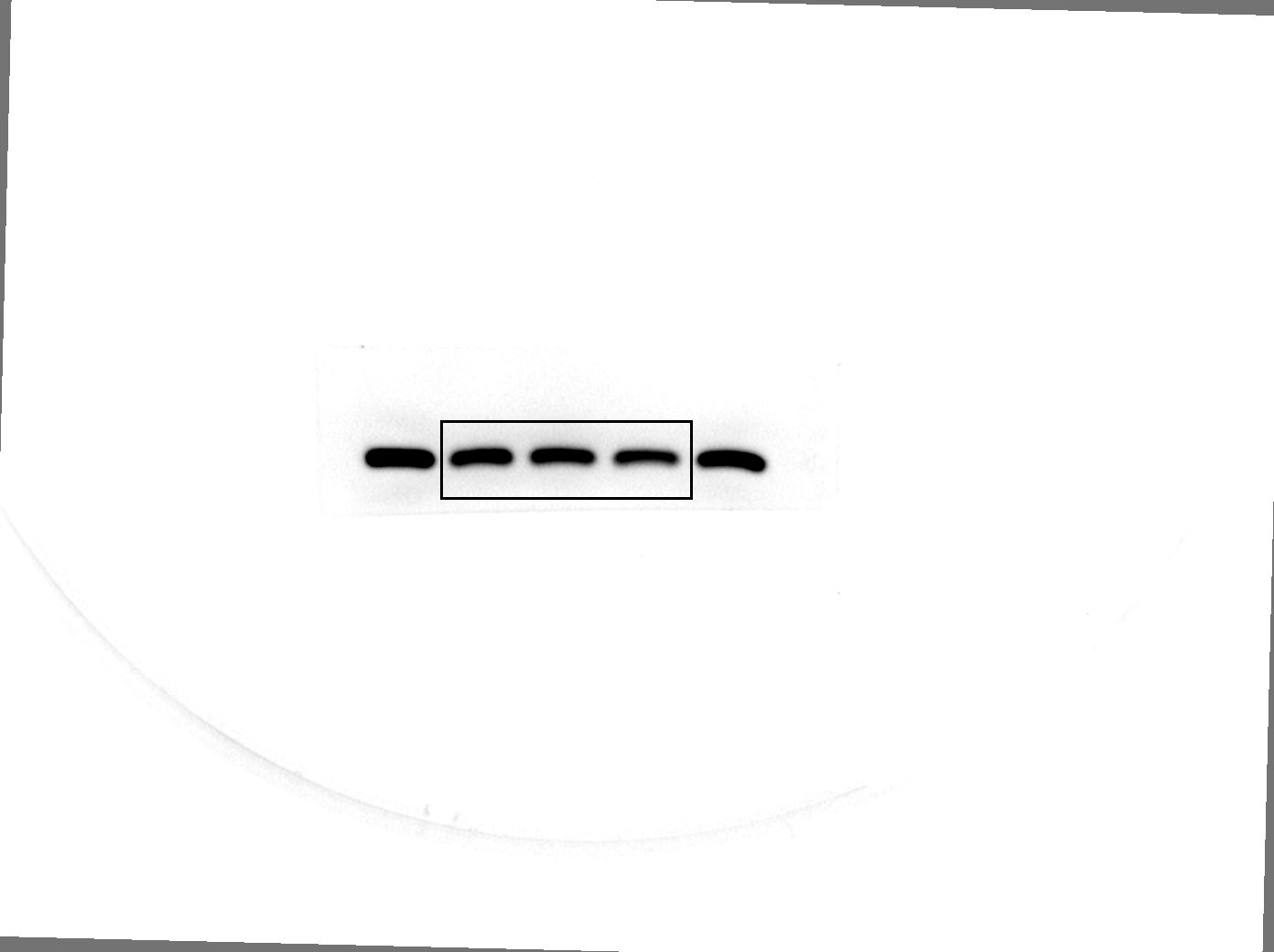

Supplement: Supplementary file 19 — Source data Fig. 4 [file 44318_2024_262_MOESM19_ESM.zip › Figure 4/4A/IB-Tubulin.tif]

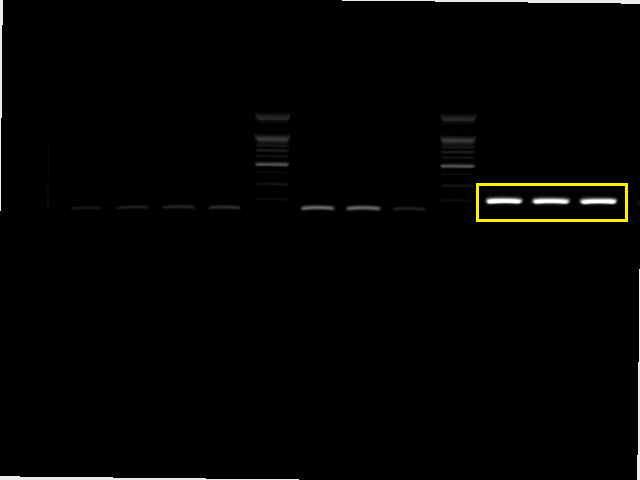

Supplement: Supplementary file 19 — Source data Fig. 4 [file 44318_2024_262_MOESM19_ESM.zip › Figure 4/4A/PCR-E22-E23.tif]

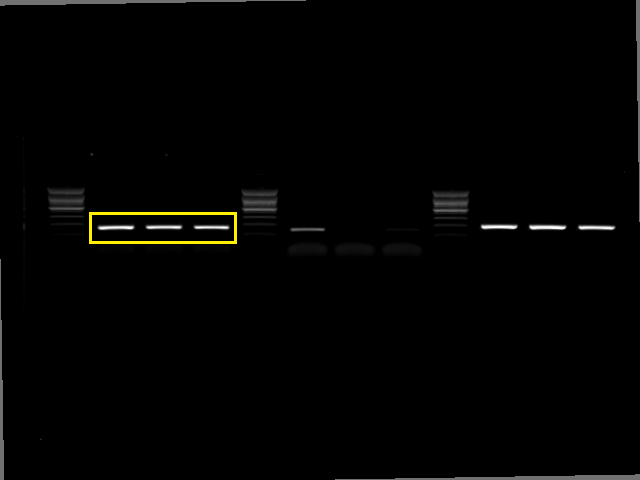

Supplement: Supplementary file 19 — Source data Fig. 4 [file 44318_2024_262_MOESM19_ESM.zip › Figure 4/4A/PCR-E25-E26.tif]

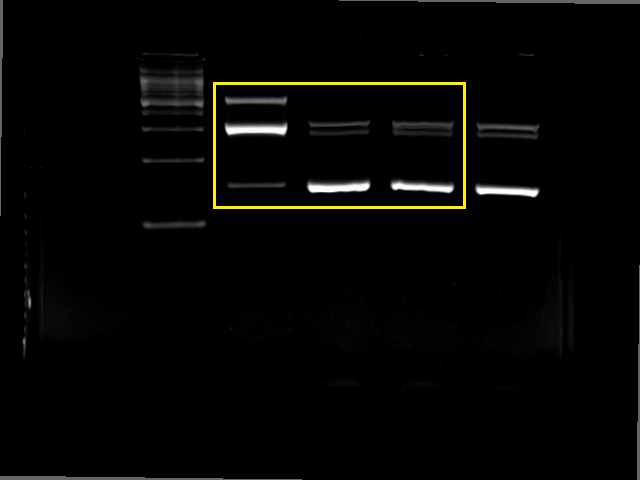

Supplement: Supplementary file 19 — Source data Fig. 4 [file 44318_2024_262_MOESM19_ESM.zip › Figure 4/4A/PCR-E25-E28.tif]

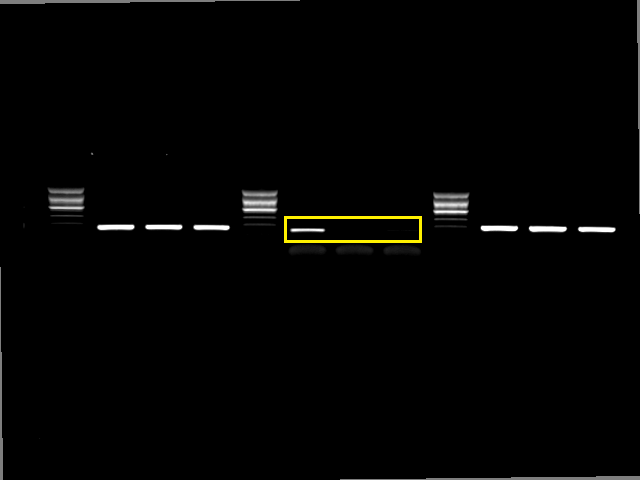

Supplement: Supplementary file 19 — Source data Fig. 4 [file 44318_2024_262_MOESM19_ESM.zip › Figure 4/4A/PCR-E27-E28.tif]

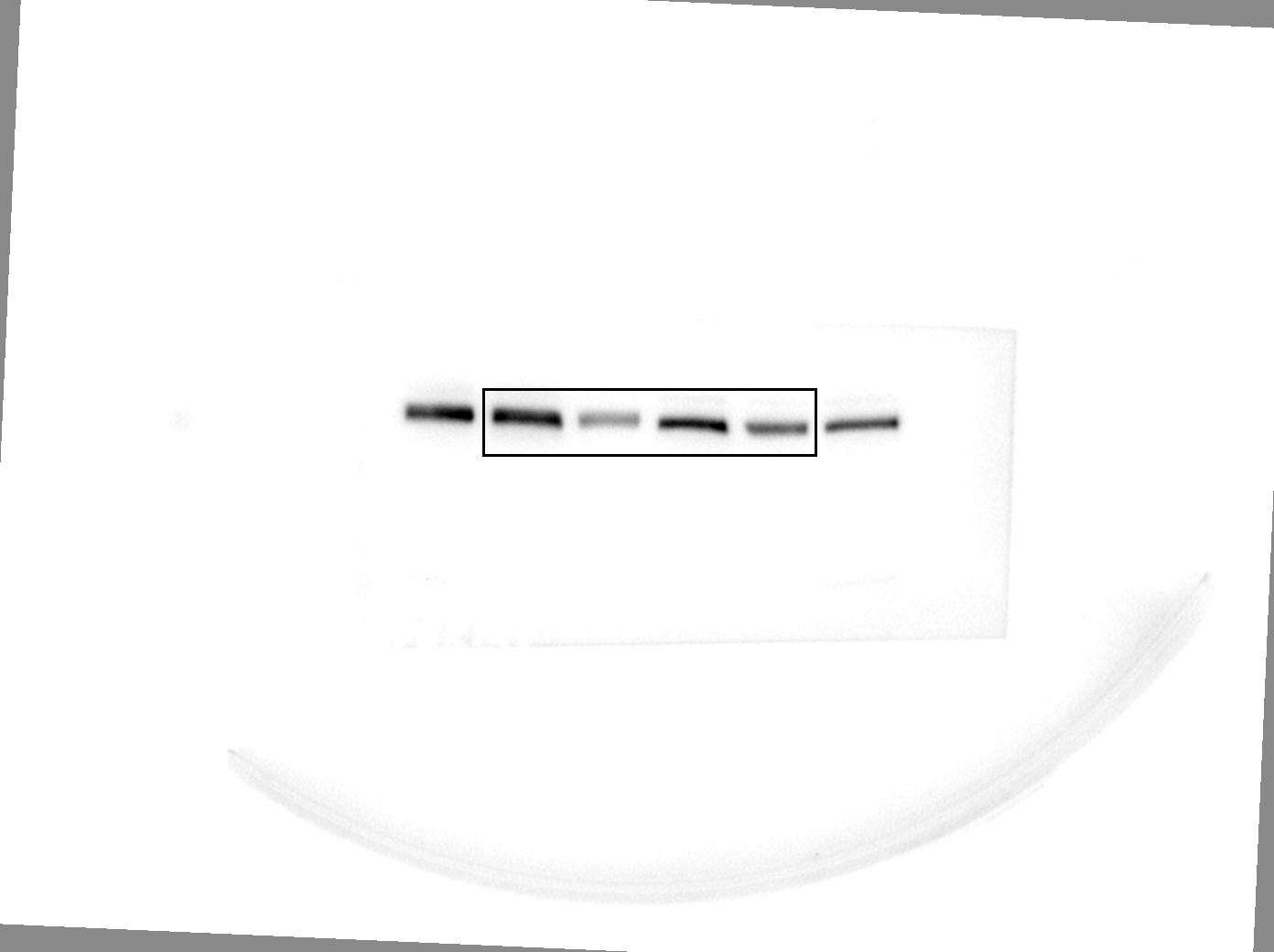

Supplement: Supplementary file 19 — Source data Fig. 4 [file 44318_2024_262_MOESM19_ESM.zip › Figure 4/4G/IB-PBRM1.tif]

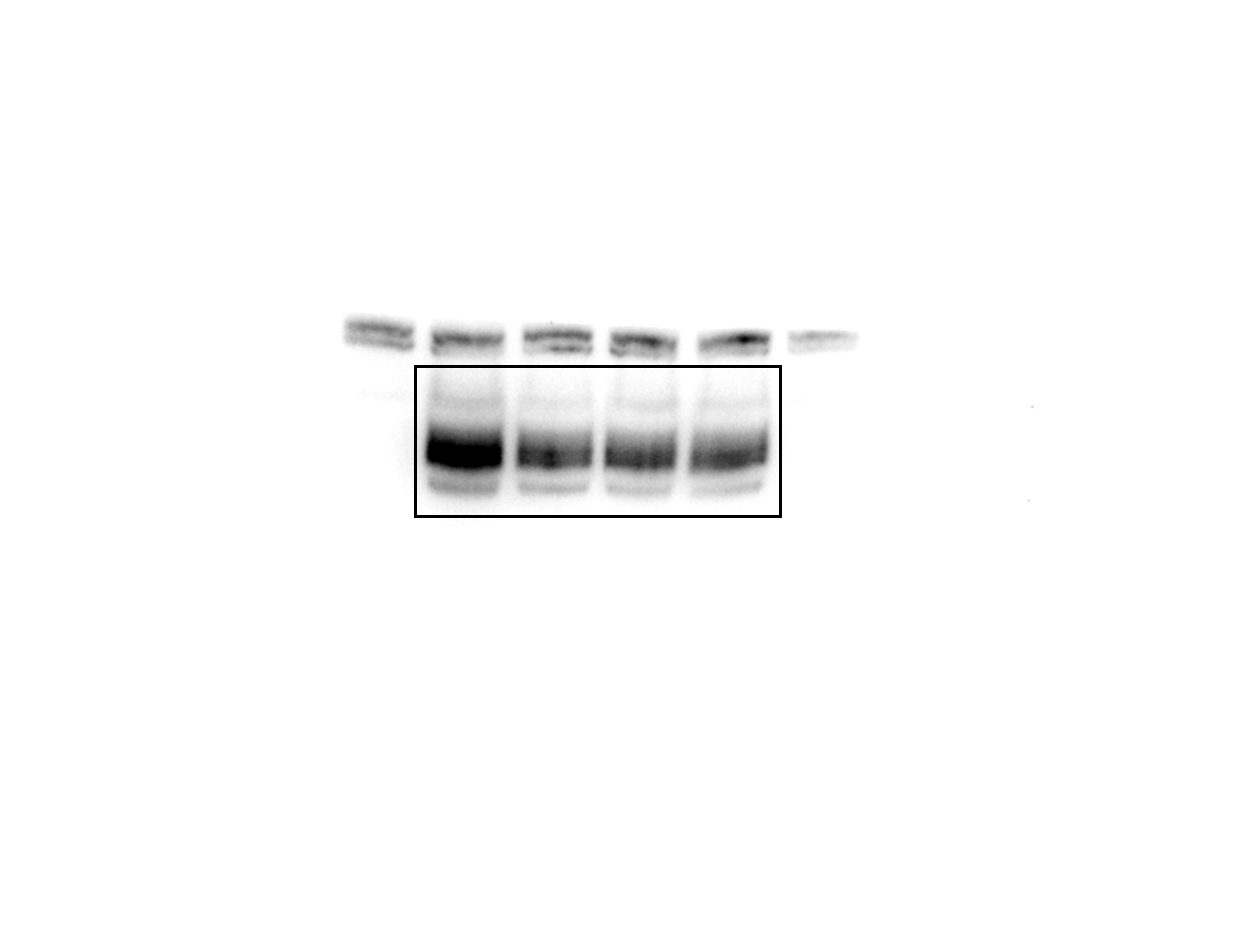

Supplement: Supplementary file 19 — Source data Fig. 4 [file 44318_2024_262_MOESM19_ESM.zip › Figure 4/4G/IB-PD-L1.tif]

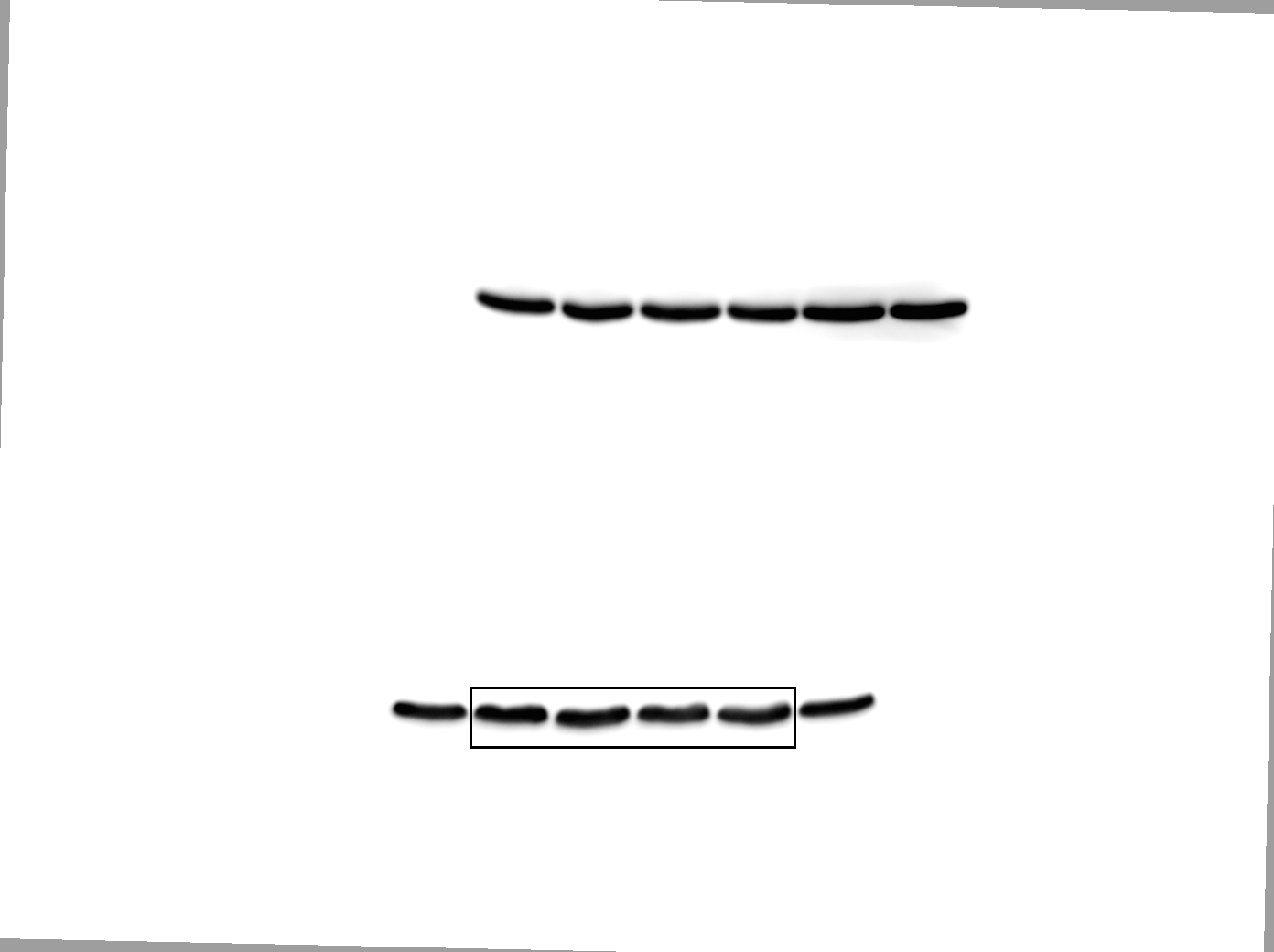

Supplement: Supplementary file 19 — Source data Fig. 4 [file 44318_2024_262_MOESM19_ESM.zip › Figure 4/4G/IB-Tubulin.tif]

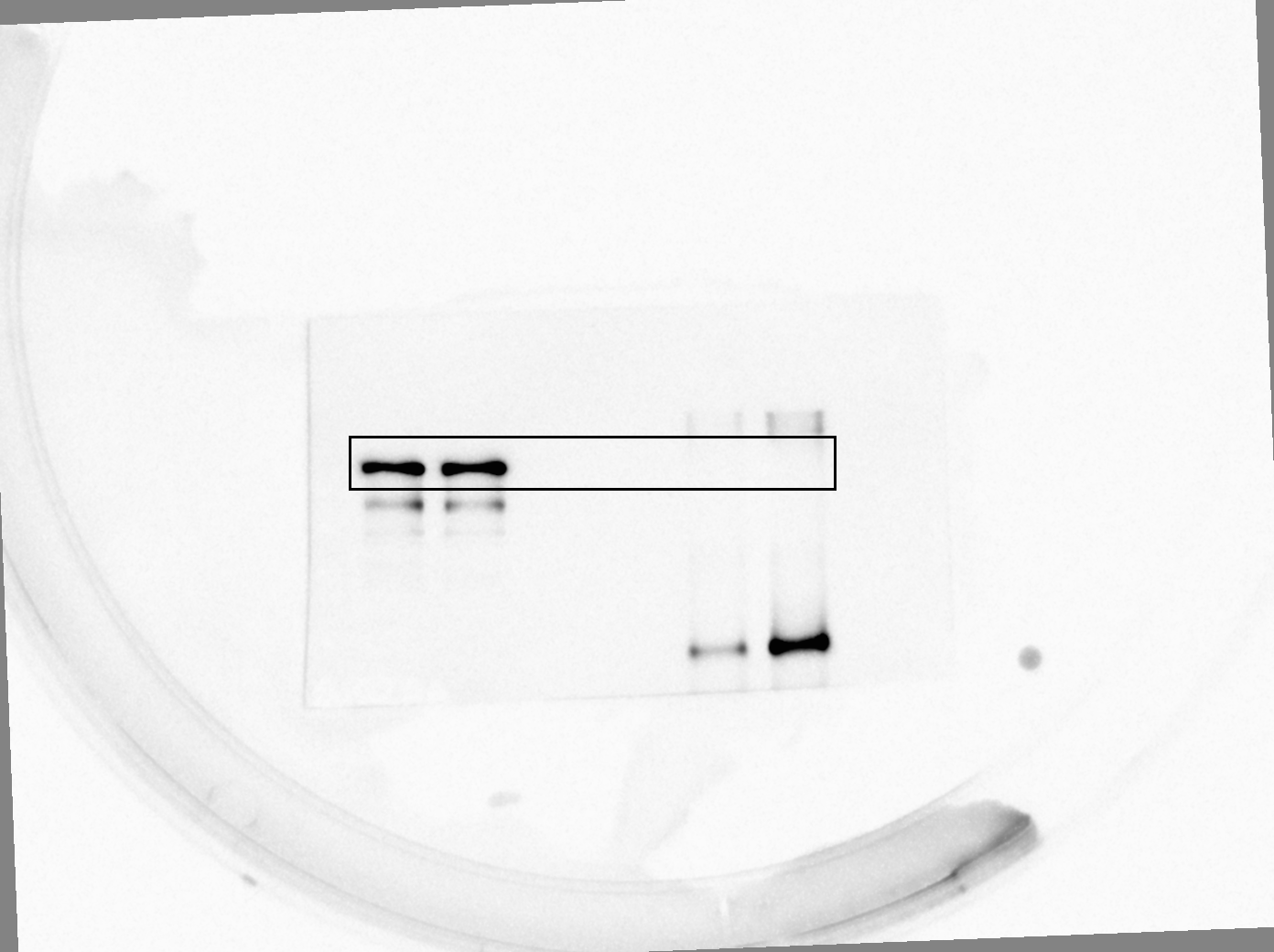

Supplement: Supplementary file 20 — Source data Fig. 5 [file 44318_2024_262_MOESM20_ESM.zip › Figure 5/5A/IB-ARID1A.tif]

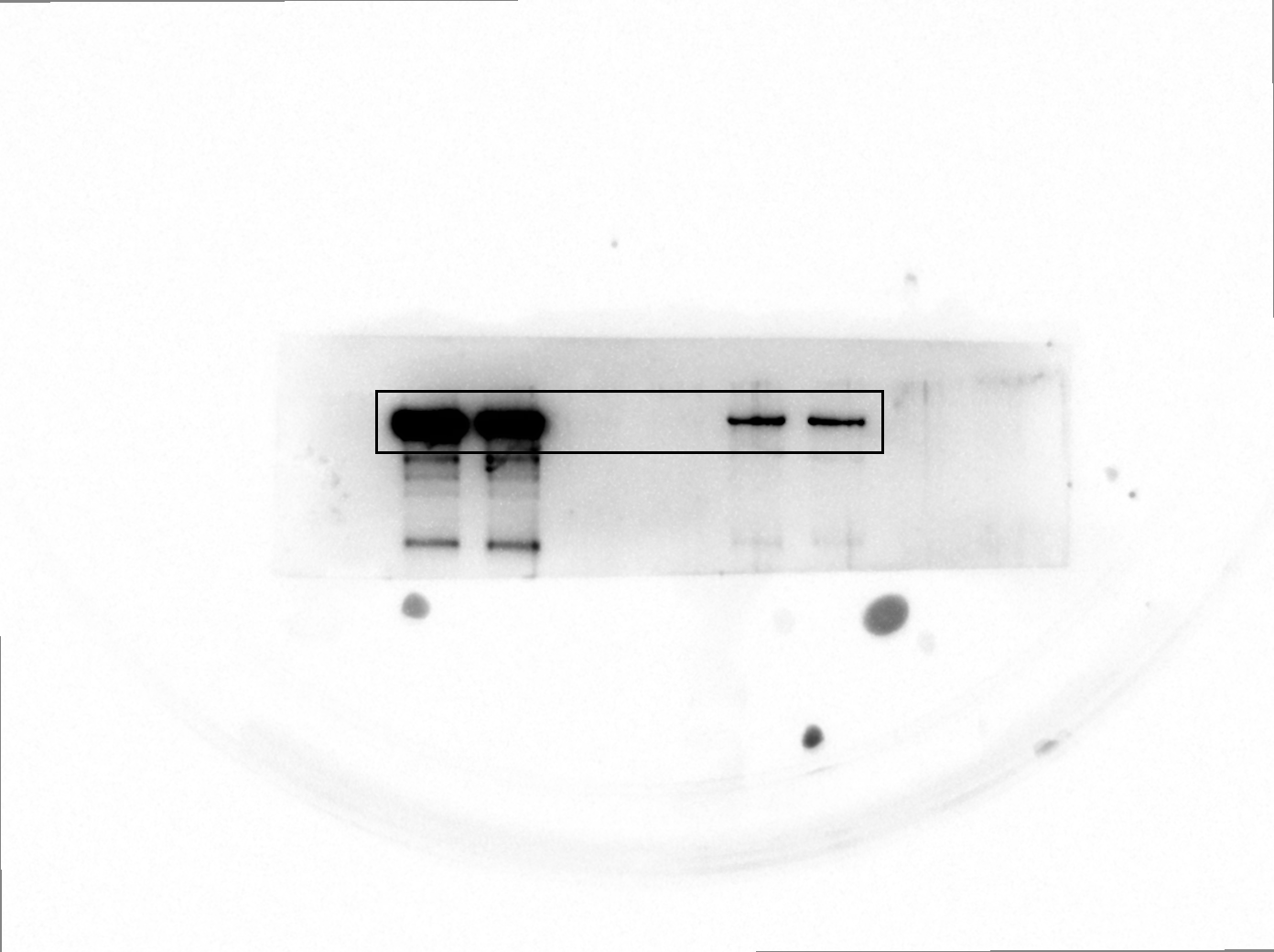

Supplement: Supplementary file 20 — Source data Fig. 5 [file 44318_2024_262_MOESM20_ESM.zip › Figure 5/5A/IB-ARID2.tif]

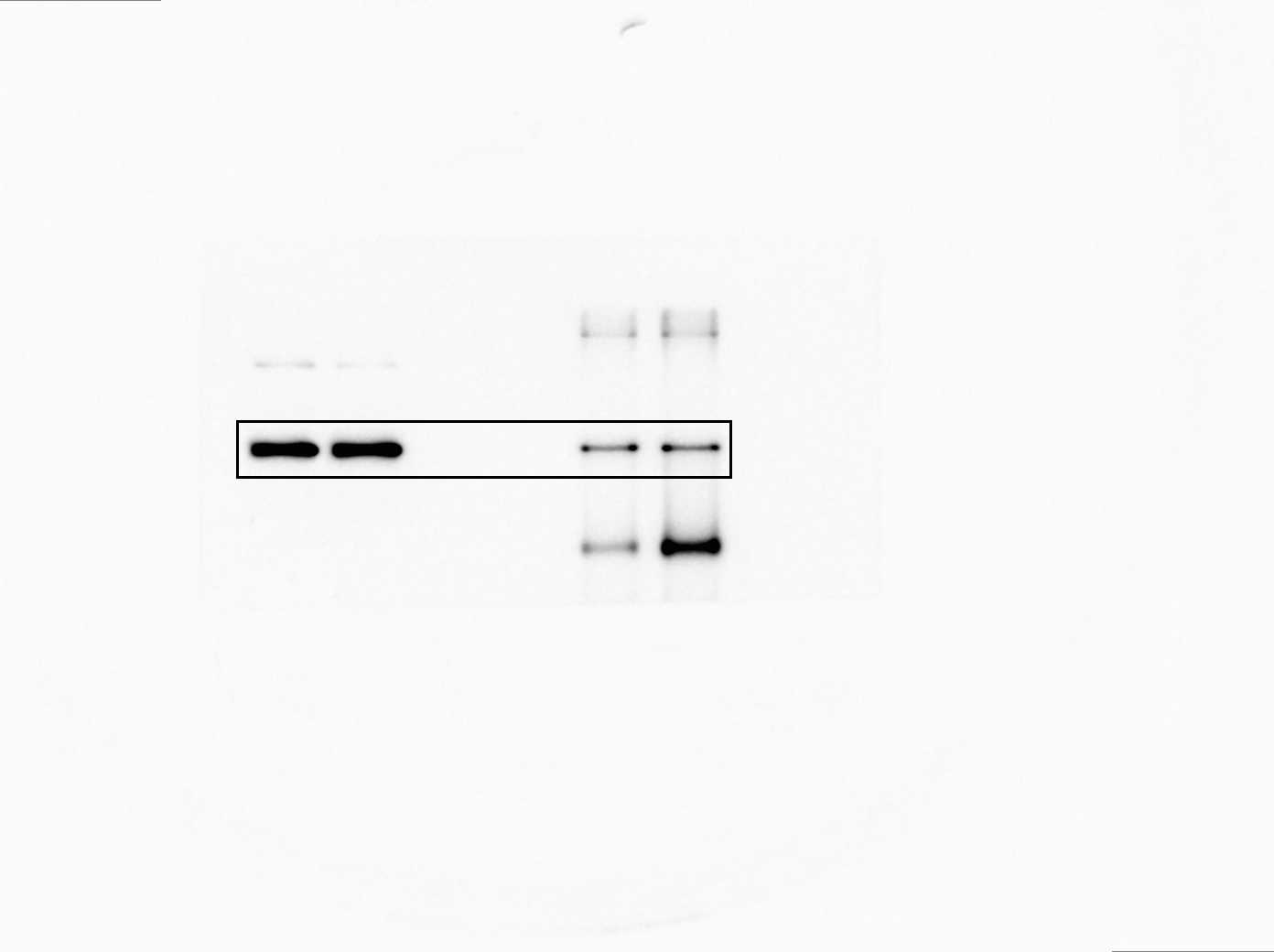

Supplement: Supplementary file 20 — Source data Fig. 5 [file 44318_2024_262_MOESM20_ESM.zip › Figure 5/5A/IB-BAF155.tif]

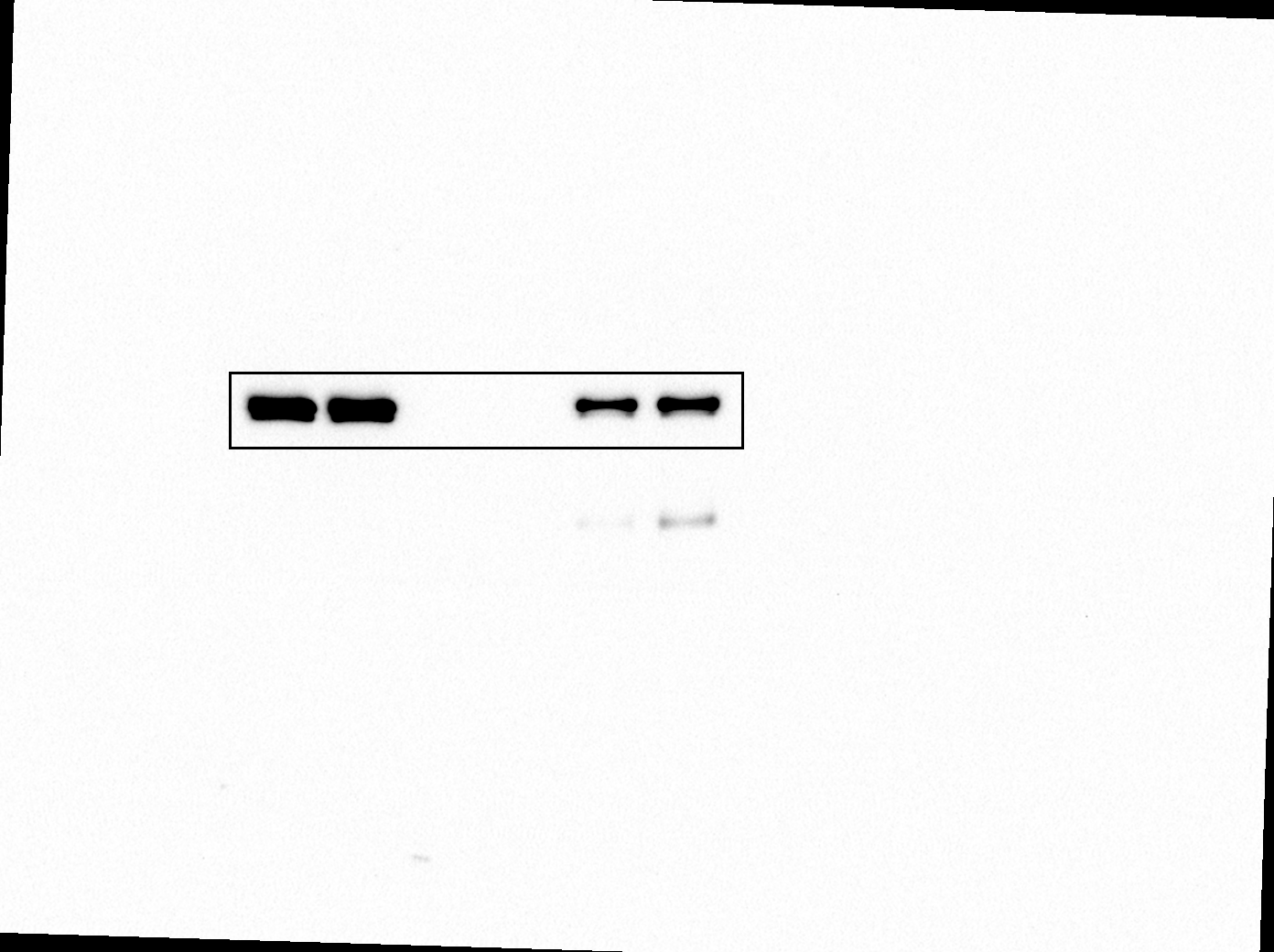

Supplement: Supplementary file 20 — Source data Fig. 5 [file 44318_2024_262_MOESM20_ESM.zip › Figure 5/5A/IB-BAF170.tif]

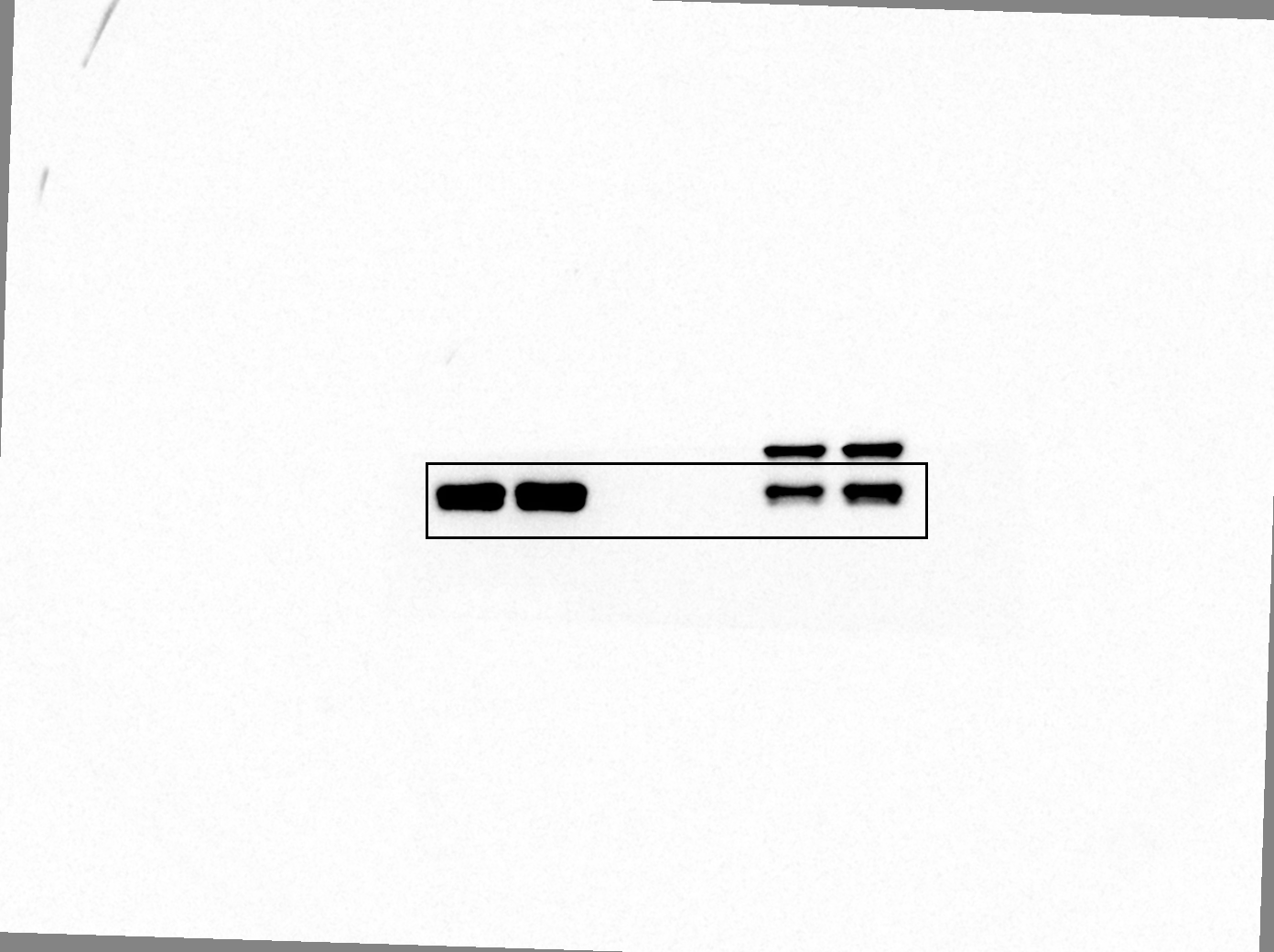

Supplement: Supplementary file 20 — Source data Fig. 5 [file 44318_2024_262_MOESM20_ESM.zip › Figure 5/5A/IB-BAF47.tif]

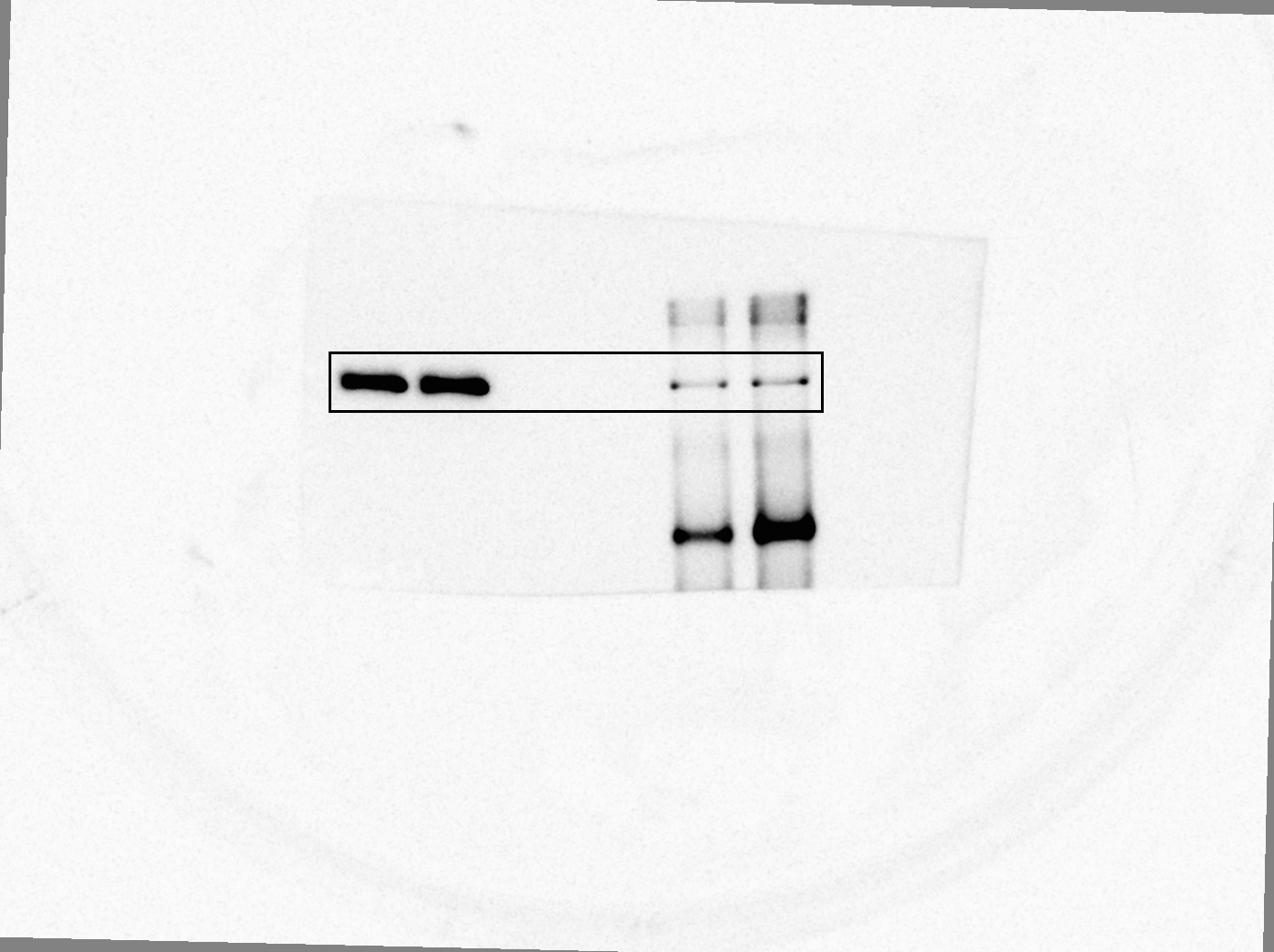

Supplement: Supplementary file 20 — Source data Fig. 5 [file 44318_2024_262_MOESM20_ESM.zip › Figure 5/5A/IB-BRG1.tif]

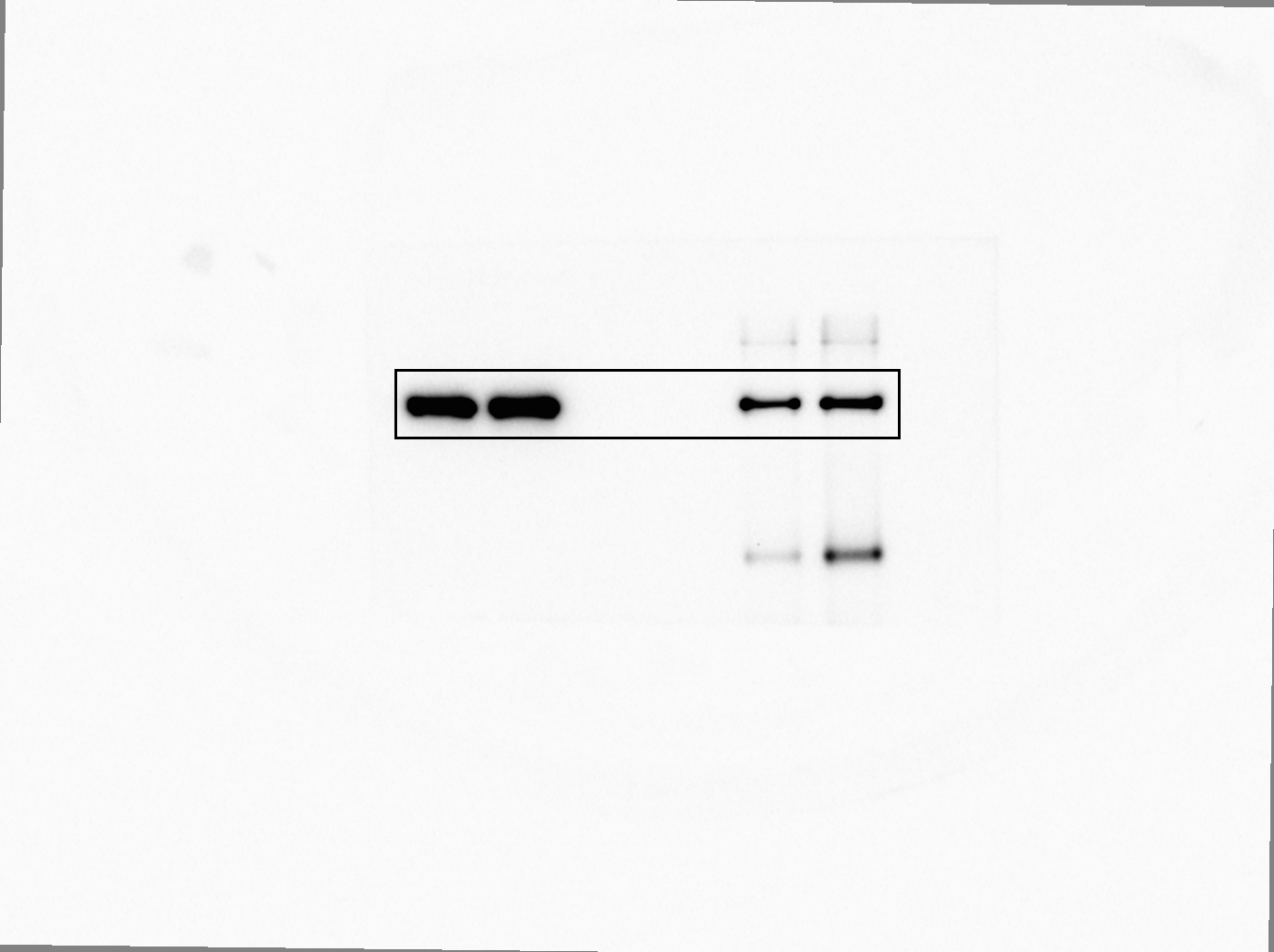

Supplement: Supplementary file 20 — Source data Fig. 5 [file 44318_2024_262_MOESM20_ESM.zip › Figure 5/5A/IB-BRM.tif]

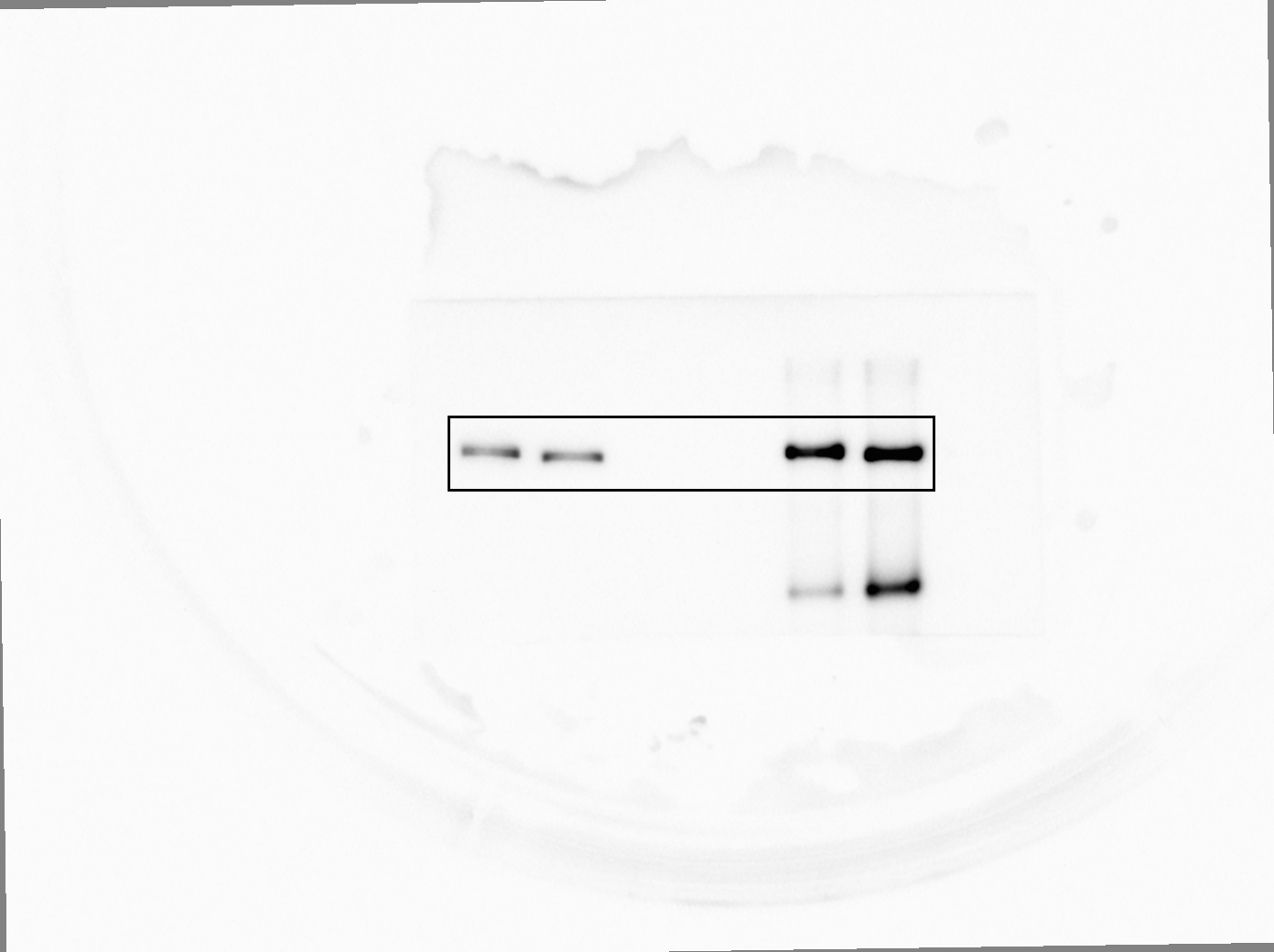

Supplement: Supplementary file 20 — Source data Fig. 5 [file 44318_2024_262_MOESM20_ESM.zip › Figure 5/5A/IB-PBRM1.tif]

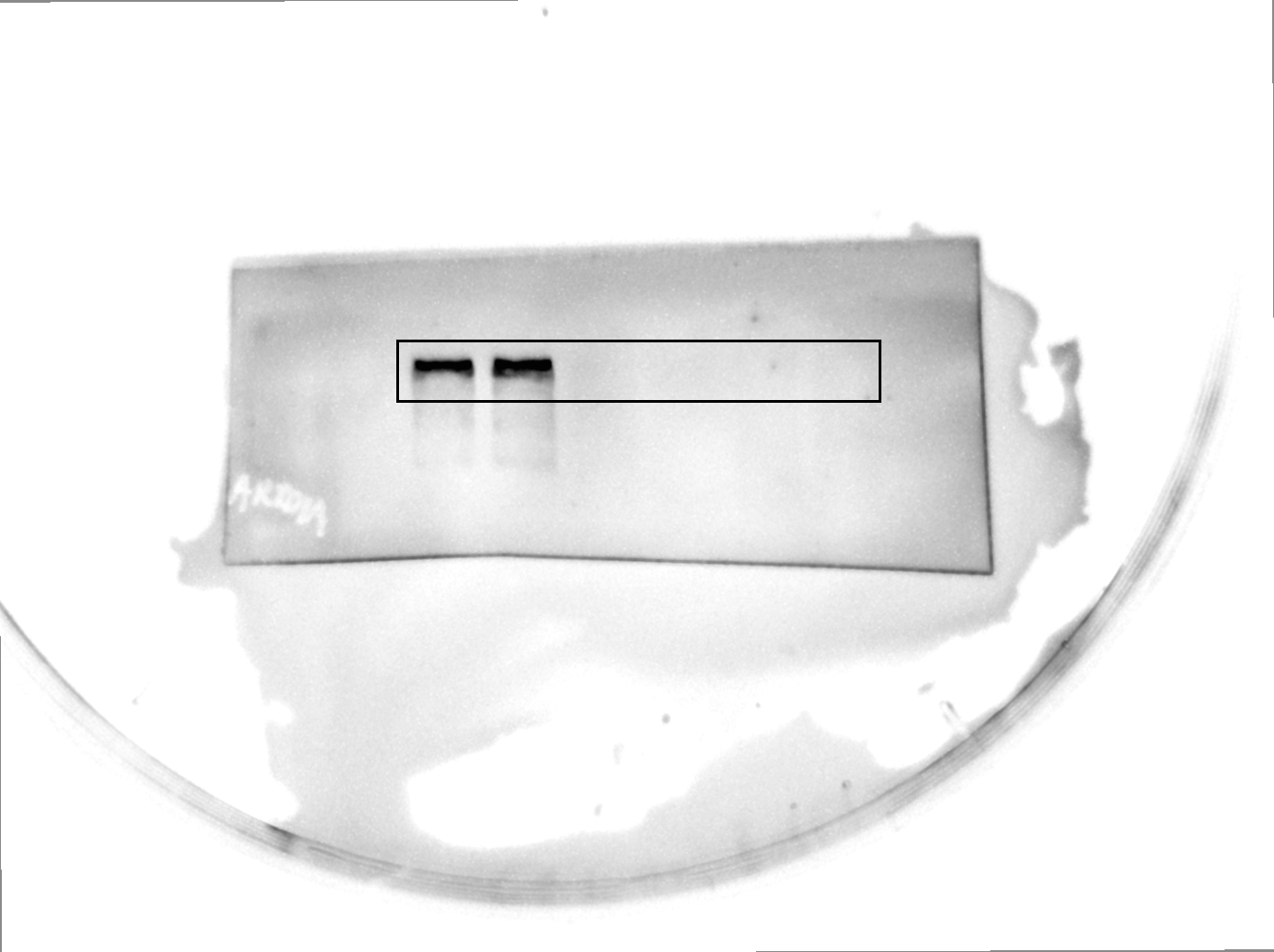

Supplement: Supplementary file 20 — Source data Fig. 5 [file 44318_2024_262_MOESM20_ESM.zip › Figure 5/5B/IB-ARID1A.tif]

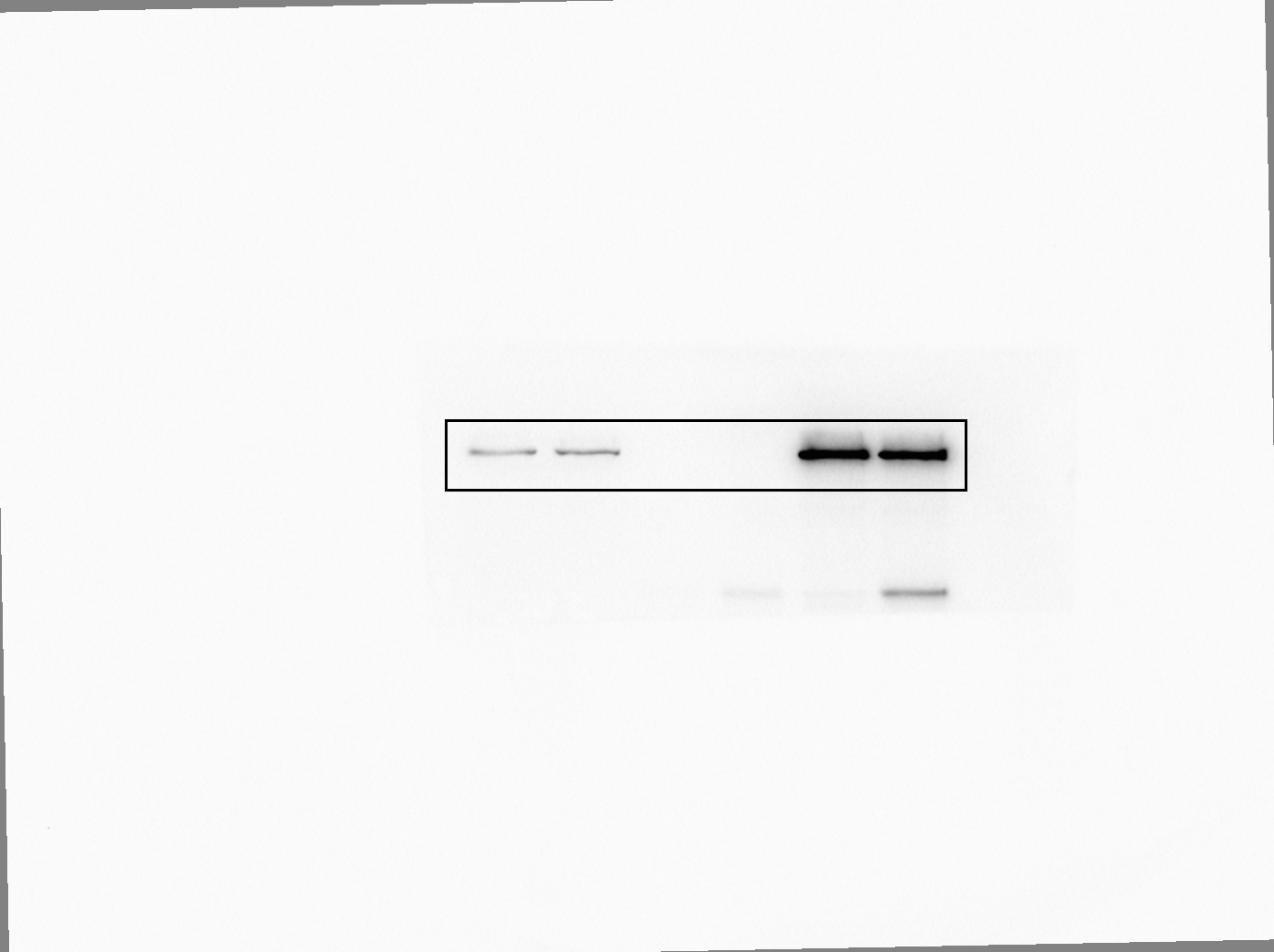

Supplement: Supplementary file 20 — Source data Fig. 5 [file 44318_2024_262_MOESM20_ESM.zip › Figure 5/5B/IB-ARID2.tif]

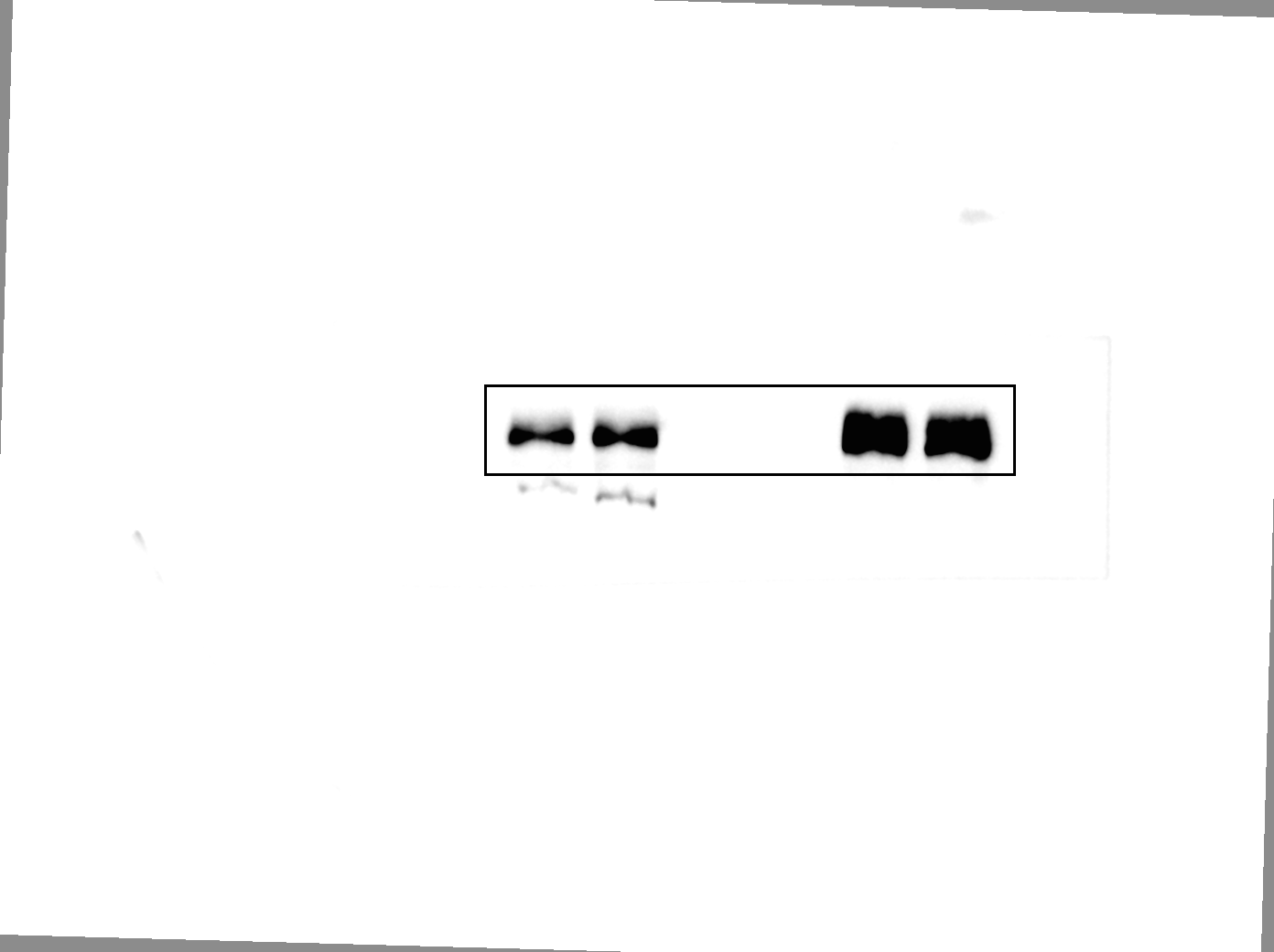

Supplement: Supplementary file 20 — Source data Fig. 5 [file 44318_2024_262_MOESM20_ESM.zip › Figure 5/5B/IB-BRM.tif]

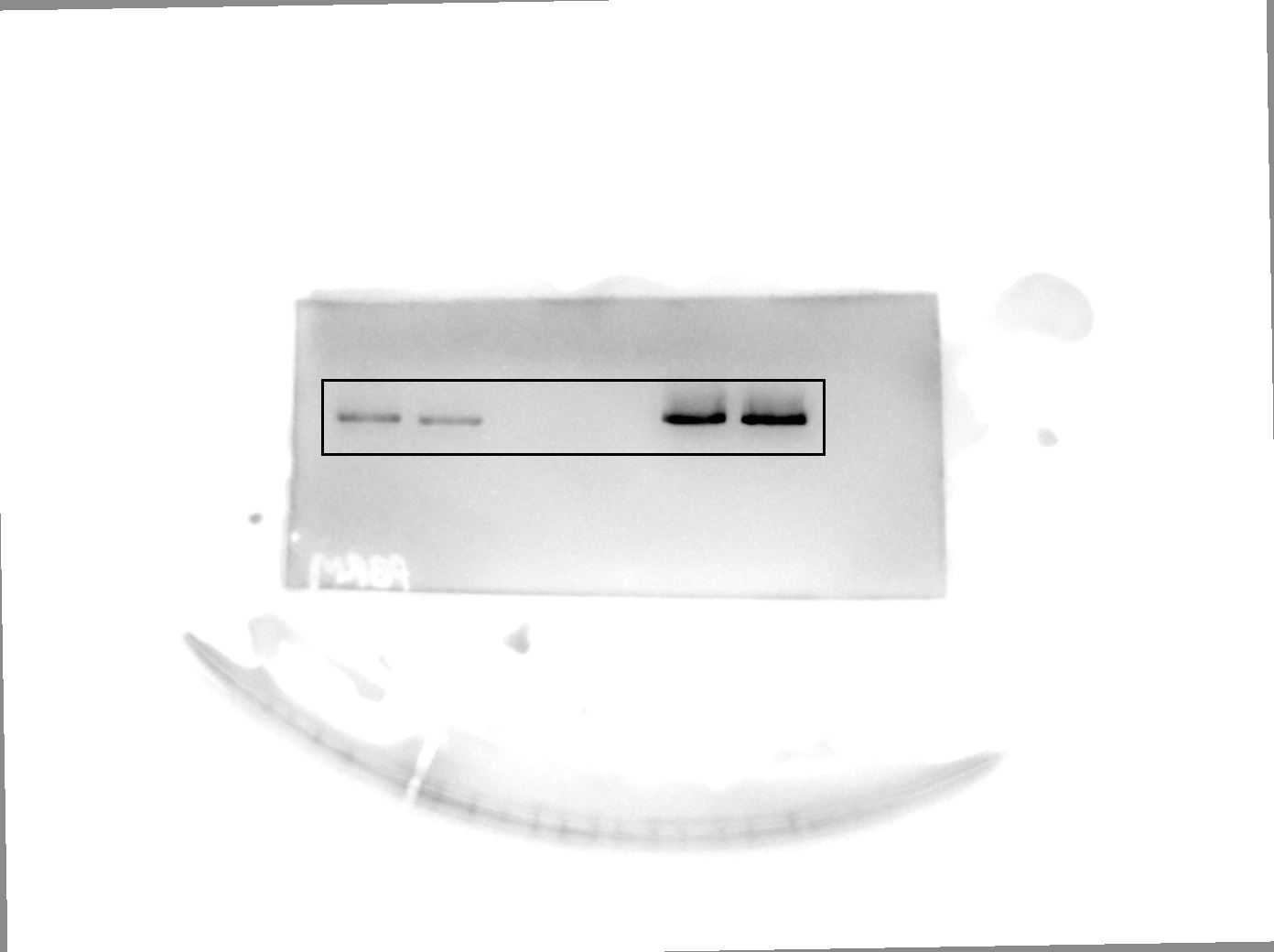

Supplement: Supplementary file 20 — Source data Fig. 5 [file 44318_2024_262_MOESM20_ESM.zip › Figure 5/5B/IB-PBRM1.tif]
