# Supplementary material for: Alternative splicing of PBRM1 mediates resistance to PD-1 blockade therapy in renal cancer
Source: EMBO J. 2024 Oct 7;43(22):7. doi: 10.1038/s44318-024-00262-7 (PMC11574163; doi:10.1038/s44318-024-00262-7)
Supplement: Supplementary file 23 — Expanded View Figures [file 44318_2024_262_MOESM23_ESM.pdf]

Expanded View Figures

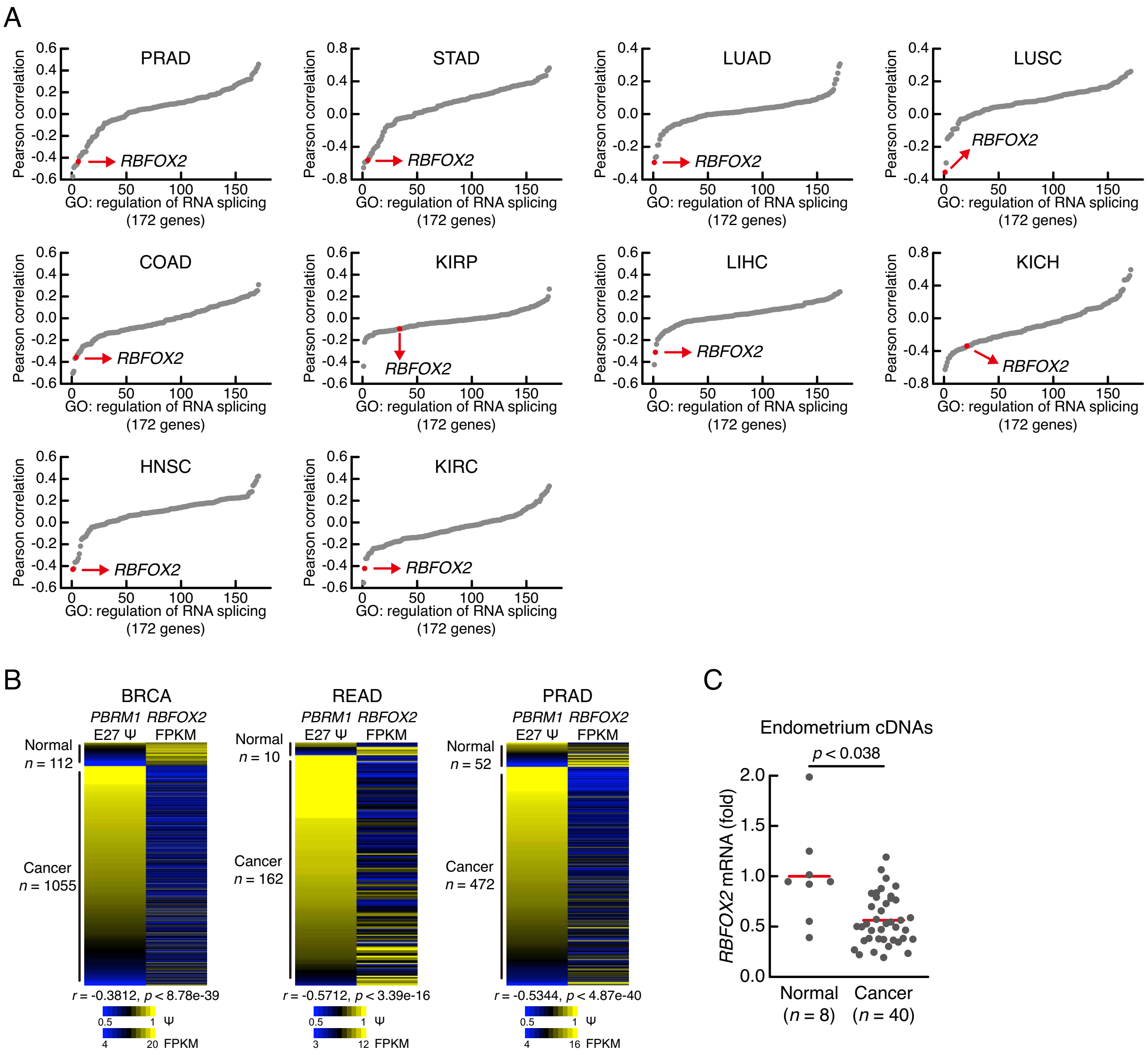

Figure EV1. *RBFOX2* mRNA levels inversely correlate with PSI values of *PBRM1* E27 in cancer tissues.

(**A**) Dot plots of splicing factors showing Pearson's correlation coefficients between FPKM values of the splicing factors and PSI values of *PBRM1* E27 in individual TCGA cancer tissues. *RBFOX2* is indicated with a red dot. (**B**) Heatmaps showing the inverse correlation between the PSI values of *PBRM1* E27 and mRNA levels of *RBFOX2* in BRCA, READ, and PRAD. Pearson's correlation coefficients and *p*-values are shown at the bottom of the heatmap. *n*, number of samples; Ψ, PSI. (**C**) *RBFOX2* mRNA levels in endometrium cDNAs. The red lines represent the means. *n*, number of samples. *p*-values were calculated using a two-tailed Student's *t*-test. Source data are available online for this figure.

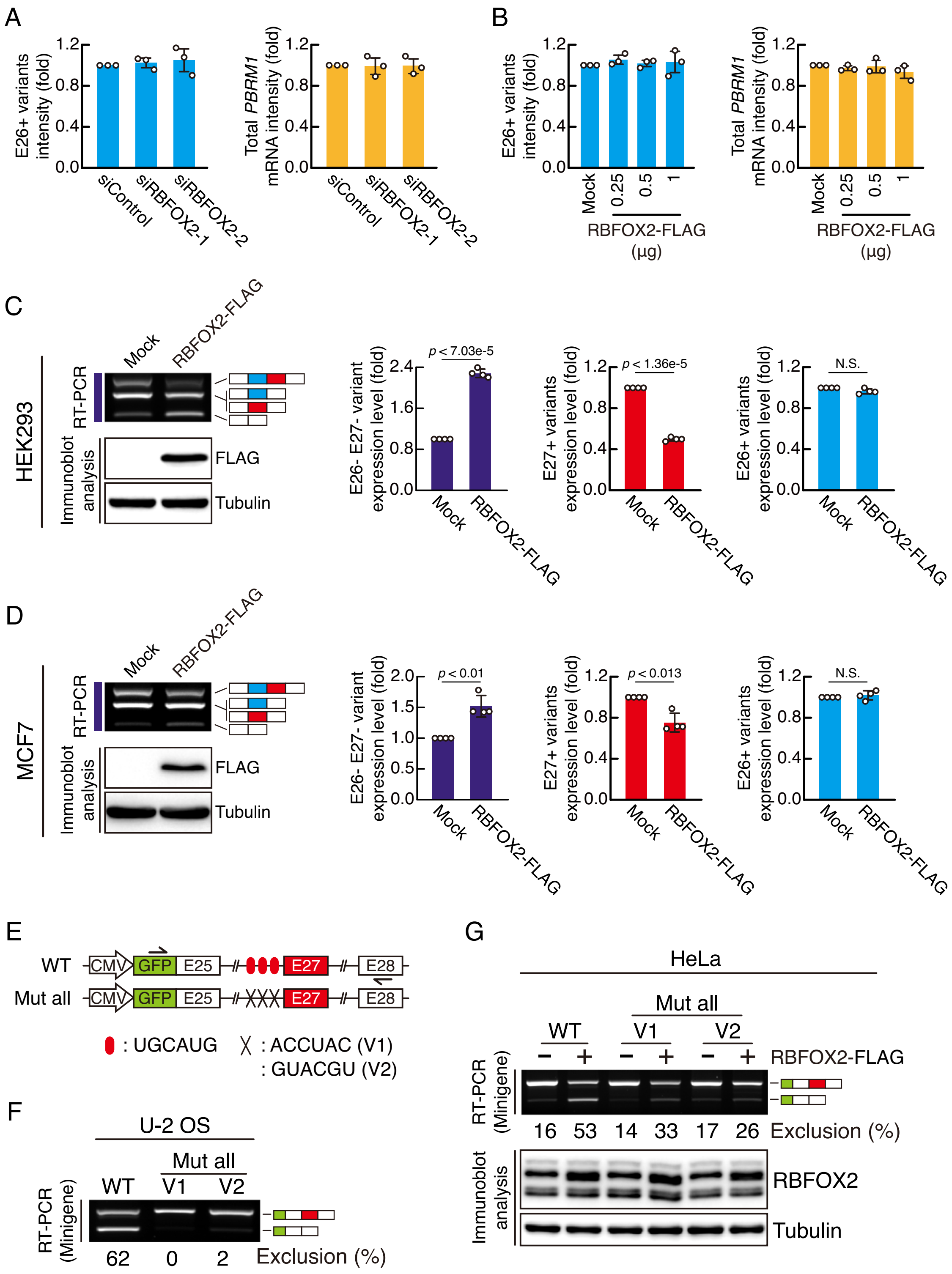

Figure EV2. RBFOX2 represses E27 inclusion in *PBRM1* mRNA.

(**A**, **B**) Quantification of RT-PCR products shown in Fig. 2F, G for E26+ variants (left) and total *PBRM1* mRNA levels in *RBFOX2*-knockdown U-2 OS cells (**A**) or in RBFOX2-FLAG-overexpressed HeLa cells (**B**) ($n = 3$, biological replicates). Bars indicate mean ± SD. (**C**, **D**) AS patterns of *PBRM1* in RBFOX2 overexpressed cells. HEK293 (**C**) and MCF7 (**D**) cells were transfected with RBFOX2 expression vectors for 36 h, followed by RT-PCR (top left), immunoblot (bottom left), and qRT-PCR (right) analyses ($n = 4$, biological replicates). Bars indicate mean ± SD. *p*-values were calculated using a two-tailed Student's *t*-test. N.S., not significant. (**E**) Schematic representation of *PBRM1* minigene constructs. (**F**) AS pattern of *PBRM1* minigene constructs in U-2 OS cells. (**G**) RT-PCR (top) and immunoblot (bottom) analyses for the AS pattern of *PBRM1* minigene transcripts by RBFOX2-FLAG overexpression in HeLa cells. Source data are available online for this figure.

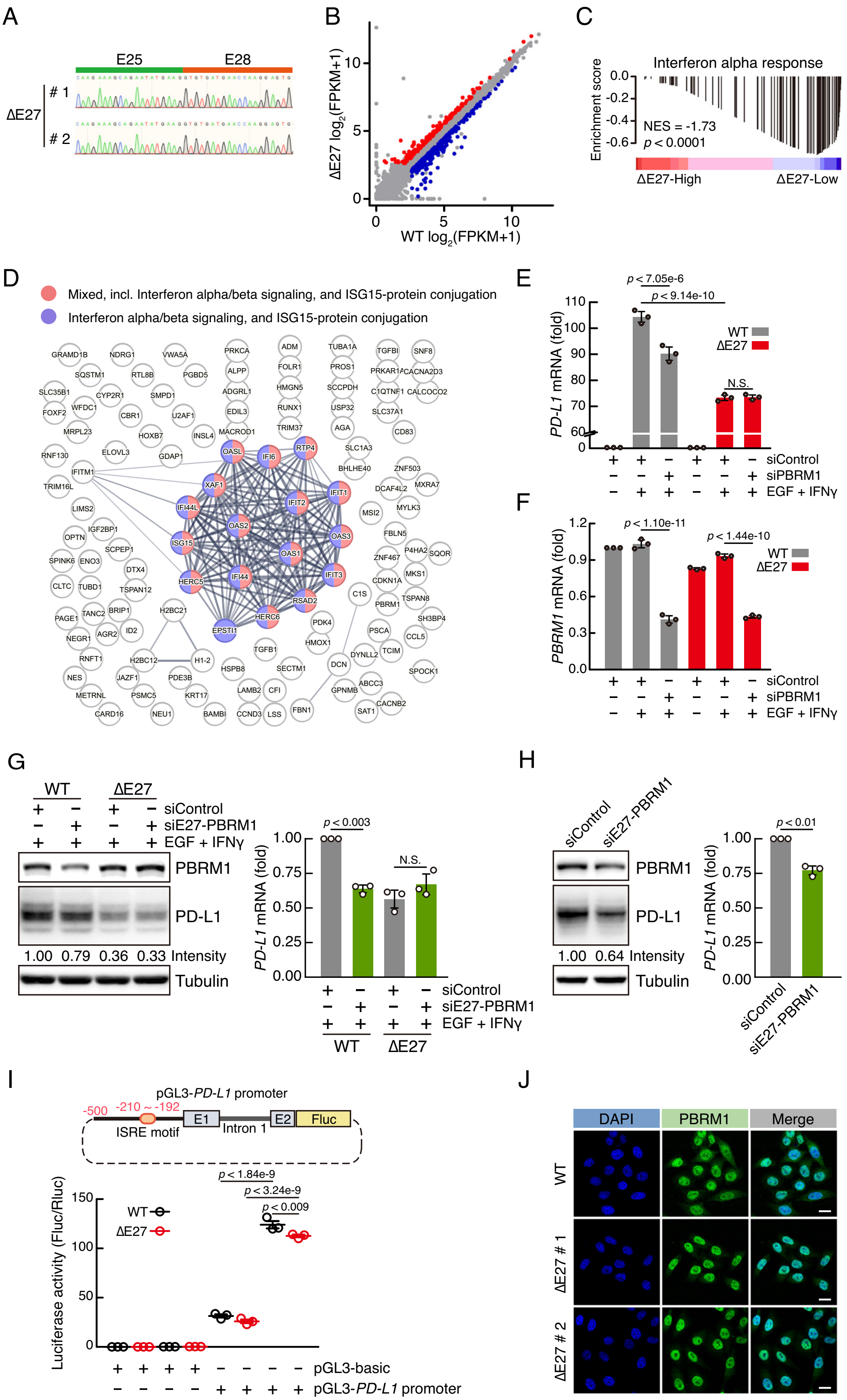

A
E25
E28
ΔE27
1
2
B
ΔE27 log2(FPKM+1)
WT log2(FPKM+1)
C
Interferon alpha response
Enrichment score
NES = -1.73
p < 0.0001
ΔE27-High
ΔE27-Low
D
Mixed, incl. Interferon alpha/beta signaling, and ISG15-protein conjugation
Interferon alpha/beta signaling, and ISG15-protein conjugation
E
PD-L1 mRNA (fold)
p < 7.05e-6
p < 9.14e-10
N.S.
WT
ΔE27
siControl
siPBRM1
EGF + IFNγ
F
PBRM1 mRNA (fold)
p < 1.10e-11
p < 1.44e-10
G
WT
ΔE27
siControl
siE27-PBRM1
EGF + IFNγ
PBRM1
PD-L1
1.00 0.79 0.36 0.33 Intensity
Tubulin
p < 0.003
N.S.
H
siControl
siE27-PBRM1
1.00 0.64 Intensity
p < 0.01
I
pGL3-PD-L1 promoter
-500
-210 ~ -192
ISRE motif
E1
Intron 1
E2
Fluc
Luciferase activity (Fluc/Rluc)
WT
ΔE27
p < 1.84e-9
p < 3.24e-9
p < 0.009
pGL3-basic
pGL3-PD-L1 promoter
J
DAPI
PBRM1
Merge
WT
ΔE27 # 1
ΔE27 # 2

Figure EV3. PBRM1 differentially affects gene expression based on E27 AS.

(**A**) Sanger sequencing with RT-PCR products of skipping variant, lacking E26 and E27, of *PBRM1* expressed in ΔE27 HeLa cell lines. (**B**) Scatter plot of RNA sequencing data displaying upregulated (red) and downregulated (blue) genes in ΔE27 HeLa cells compared to those in WT HeLa cells. (**C**) GSEA plot of IFNα-responsive genes in all gene transcripts of ΔE27 HeLa cells versus WT HeLa cells. The nominal *p*-value is presented. (**D**) STRING protein-protein network analysis of the top 120 downregulated genes in ΔE27 HeLa cells. The lines indicate the co-expression network between proteins and the line thickness indicates the strength of data support. (**E**, **F**) qRT-PCR analysis of *PD-L1* (**E**) or *PBRM1* (**F**) mRNA levels. HeLa cells were treated with 20 ng/ml EGF and 100 ng/ml IFNγ for 12 h following transfection with siPBRM1 for 36 h ($n = 3$, biological replicates). Bars indicate mean ± SD. *p*-values were calculated using one-way ANOVA with Dunnett's multiple comparison test. (**G**, **H**) Immunoblot (left) and qRT-PCR (right) analyses confirming PD-L1 expression levels following knockdown of the E27-included PBRM1 isoforms using siRNA targeting PBRM1 E27 sequences (siE27-PBRM1) in HeLa (**G**) or MDA-MB-231 cells (**H**). $n = 3$, biological replicates. Bars indicate mean ± SD. *p*-values were calculated using two-tailed Student's *t*-test. N.S., not significant. (**I**) *PD-L1* promoter reporter assay in WT and ΔE27 HeLa cells. The schematic shows the *PD-L1* promoter reporter vector (top), and the graph presents the results of firefly luciferase (Fluc) activities normalised to renilla luciferase (Rluc) activities (bottom). $n = 3$, biological replicates. Lines indicate the mean. *p*-values were calculated using one-way ANOVA with Bonferroni's multiple comparison test. ISRE, Interferon-sensitive response element. (**J**) Immunofluorescence analysis of PBRM1 (green) and DAPI (nuclei; blue) in WT and ΔE27 HeLa cells. Scale bars, 20 μm. Source data are available online for this figure.

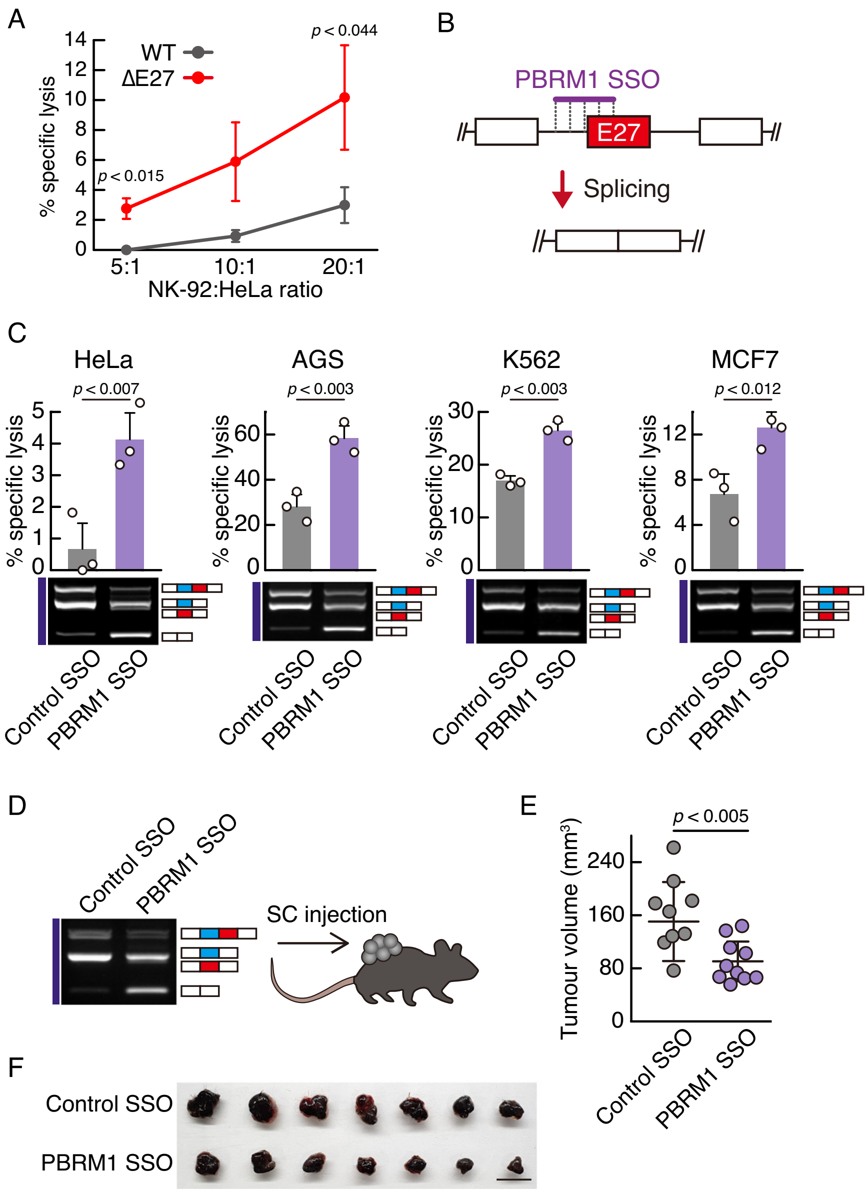

Figure EV4. SSO-induced E27 exclusion of *PBRM1* suppresses cancer immune evasion.

(**A**) NK-92 cell cytotoxicity against ΔE27 HeLa cells. HeLa cells were co-cultured with NK-92 cells for 7 h ($n = 3$, biological replicates). *p*-values were calculated using one-tailed Student's *t*-test. (**B**) Design of PBRM1 SSO for induction of E27 exclusion in *PBRM1*. (**C**) NK-92 cell cytotoxicity against PBRM1 SSO-transfected cancer cells (top). NK-92 cells were co-cultured with cancer cells at an effector:target ratio of 20:1 for 7 h ($n = 3$, biological replicates). SSO-induced *PBRM1* E27 exclusion was confirmed using RT-PCR (bottom). Bars indicate mean ± SD. *p*-values were calculated using one-tailed Student's *t*-test. (**D**) RT-PCR analysis confirmed *PBRM1* E27 exclusion by PBRM1 SSO transfection into B16-F10 cells used for subcutaneous injection into mice. (**E**, **F**) Tumour volume (**E**) and representative images of tumour (**F**) from C57BL/6 mice injected subcutaneously with control SSO- ($n = 9$, individual mice) or PBRM1 SSO-transfected B16-F10 cells ($n = 10$, individual mice) on day 9. The *p*-value was calculated using two-tailed Student's *t*-test. Scale bar, 1 cm. Source data are available online for this figure.

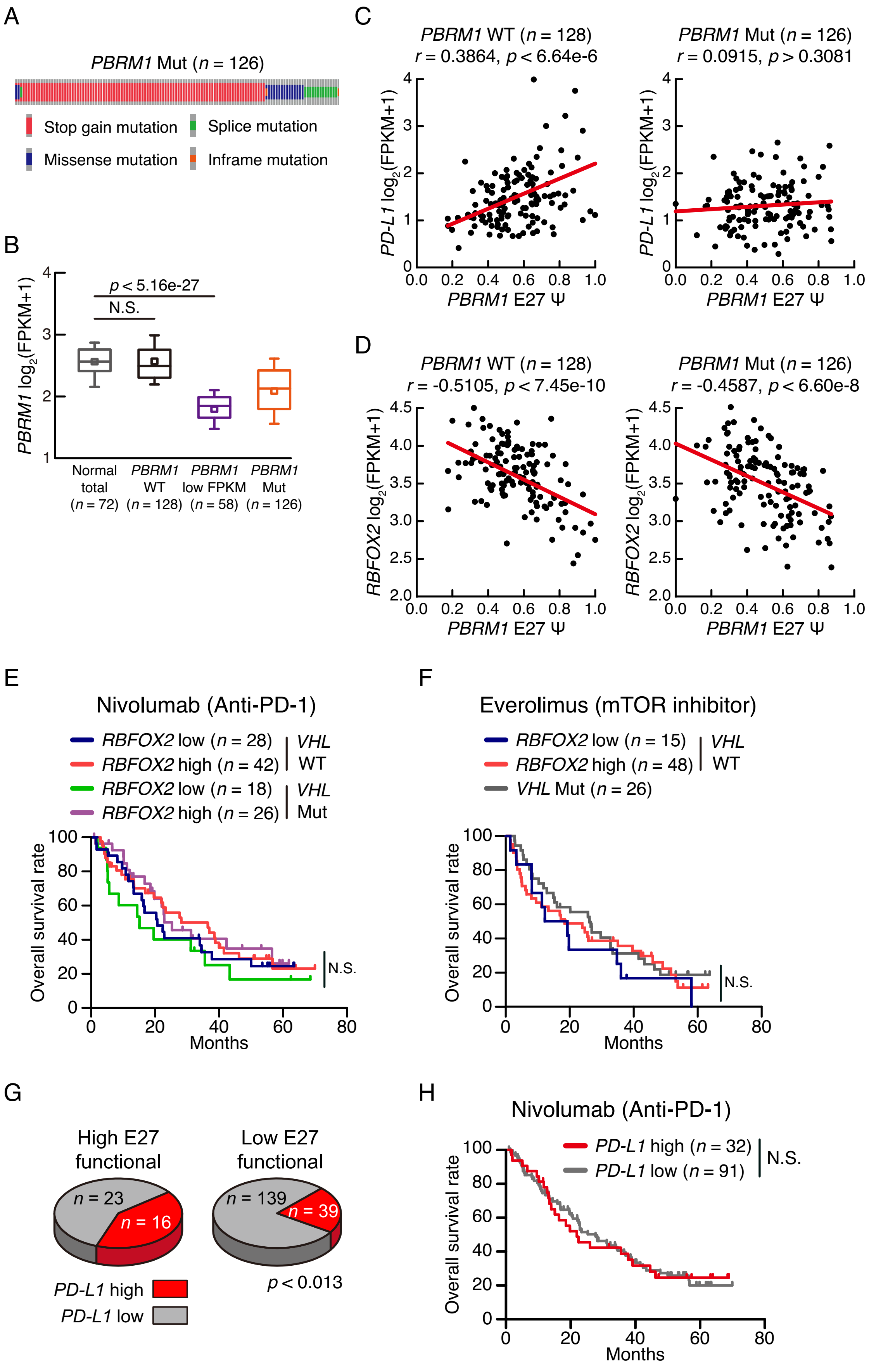
A
PBRM1 Mut (n = 126)
Stop gain mutation
Splice mutation
Missense mutation
Inframe mutation
B
p < 5.16e-27
N.S.
PBRM1 log2(FPKM+1)
Normal total (n = 72)
PBRM1 WT (n = 128)
PBRM1 low FPKM (n = 58)
PBRM1 Mut (n = 126)
C
PBRM1 WT (n = 128)
r = 0.3864, p < 6.64e-6
PBRM1 Mut (n = 126)
r = 0.0915, p > 0.3081
PD-L1 log2(FPKM+1)
PBRM1 E27 Ψ
D
PBRM1 WT (n = 128)
r = -0.5105, p < 7.45e-10
PBRM1 Mut (n = 126)
r = -0.4587, p < 6.60e-8
RBFOX2 log2(FPKM+1)
PBRM1 E27 Ψ
E
Nivolumab (Anti-PD-1)
RBFOX2 low (n = 28)
RBFOX2 high (n = 42)
VHL WT
RBFOX2 low (n = 18)
RBFOX2 high (n = 26)
VHL Mut
Overall survival rate
Months
N.S.
F
Everolimus (mTOR inhibitor)
RBFOX2 low (n = 15)
RBFOX2 high (n = 48)
VHL WT
VHL Mut (n = 26)
Overall survival rate
Months
N.S.
G
High E27 functional
n = 23
n = 16
Low E27 functional
n = 139
n = 39
PD-L1 high
PD-L1 low
p < 0.013
H
Nivolumab (Anti-PD-1)
PD-L1 high (n = 32)
PD-L1 low (n = 91)
N.S.
Overall survival rate
Months

Figure EV5. E27 inclusion rates in *PBRM1* correlate with *PD-L1* expression in *PBRM1* WT ccRCC but not in *PBRM1* Mut ccRCC.

(**A**) Mutation frequency in the *PBRM1* gene across patients with KIRC in the *PBRM1* Mut group. Boxes indicate cancer tissues, with colours representing the four somatic mutation statuses. (**B**) Box plot showing *PBRM1* mRNA level in normal and ccRCC tissues. Boxes represent the median, quartiles, 10th percentile, and 90th percentile. *n*, number of samples. *p*-values were calculated by the one-way ANOVA with Bonferroni's multiple comparison test. (**C**, **D**) Scatter plot of *PBRM1* E27 PSI versus *PD-L1* mRNA level (**C**) or *RBFOX2* mRNA level (**D**) in individual *PBRM1* WT ccRCC (left) or *PBRM1* Mut ccRCC (right) cancer tissues. Pearson correlation coefficients and *p*-values are presented. *n*, number of samples; Ψ, PSI. (**E**, **F**) Kaplan–Meier survival curves of patients with ccRCC treated with nivolumab (**E**) or everolimus (**F**). Patients with ccRCC possessing the non-mutant *VHL* gene and a low *VHL* mRNA level (below 12%) were not classified in the *VHL* WT group. *n*, number of samples. (**G**) Pie charts showing the ratios of ccRCC patients with high and low *PD-L1* levels in high (*RBFOX2* low and *PBRM1* WT) or low (all others) E27 functional groups. Patients with ccRCC treated with nivolumab or everolimus were classified. *n*, number of samples. The *p*-value was calculated using chi-squared test. (**H**) Kaplan–Meier survival curve of ccRCC patients treated with nivolumab, stratified by *PD-L1* expression status. *n*, number of samples. N.S., not significant.
